# LncRNA NEAT1 controls the lineage fates of BMSCs during skeletal aging by impairing mitochondrial function and pluripotency maintenance

**DOI:** 10.1038/s41418-021-00858-0

**Published:** 2021-09-08

**Authors:** Hengguo Zhang, Rongyao Xu, Bang Li, Zhili Xin, Ziji Ling, Weiwen Zhu, Xiang Li, Ping Zhang, Yu Fu, Jiyu Chen, Laikui Liu, Jie Cheng, Hongbing Jiang

**Affiliations:** 1 Jiangsu Key Laboratory of Oral Diseases, Nanjing Medical University, Nanjing, Jiangsu Province China; 2 Department of Oral and Maxillofacial Surgery, Affiliated Hospital of Stomatology, Nanjing Medical University, Nanjing, Jiangsu Province China; 3 Jiangsu Key Laboratory of Cardiovascular and Cerebrovascular Medicine, Nanjing Medical University, Nanjing, Jiangsu Province China; 4 Department of Basic Science of Stomatology, Affiliated Hospital of Stomatology, Nanjing Medical University, Nanjing, Jiangsu Province China; 5 Jiangsu Province Engineering Research Center of Stomatological Translational Medicine, Nanjing, Jiangsu Province China

**Keywords:** Gene regulation, Endocrine system and metabolic diseases

## Abstract

Aged bone marrow mesenchymal stem cells (BMSCs) exhibit aberrant self-renewal and lineage specification, which contribute to imbalanced bone-fat and progressive bone loss. In addition to known master regulators of lineage commitment, it is crucial to identify pivotal switches governing the specific differentiation fate of aged BMSCs. Here, we profiled differences in epigenetic regulation between adipogenesis and osteogenesis and identified super-enhancer associated lncRNA nuclear-enriched abundant transcript 1 (NEAT1) as a key bone-fat switch in aged BMSCs. We validated that NEAT1 with high enhancer activity was transcriptionally activated by ATF2 and directed aged BMSCs to a greater propensity to differentiate toward adipocytes than osteoblasts by mediating mitochondrial function. Furthermore, we confirmed NEAT1 as a protein-binding scaffold in which phosphorylation modification of SOX2 Ser249/250 by CDK2 impaired SOX2/OCT4 complex stability and dysregulated downstream transcription networks of pluripotency maintenance. In addition, by sponging miR-27b-3p, NEAT1 upregulated BNIP3L, BMP2K, and PPARG expression to shape mitochondrial function and osteogenic/adipogenic differentiation commitment, respectively. In extracellular communication, NEAT1 promoted CSF1 secretion from aged BMSCs and then strengthened osteoclastic differentiation by extracellular vesicle delivery. Notably, Neat1 small interfering RNA delivery induced increased bone mass in aged mice and decreased fat accumulation in the bone marrow. These findings suggest that NEAT1 regulates the lineage fates of BMSCs by orchestrating mitochondrial function and pluripotency maintenance, and might be a potential therapeutic target for skeletal aging.

## Introduction

Age-related osteoporosis is characterized by net bone loss, a proinflammatory microenvironment, and excessive adipose tissue accumulation [1–3]. Adipocyte-enriched bone marrow during skeletal aging is primarily attributed to dysfunctional self-renewal and pluripotent differentiation of bone marrow mesenchymal stem cells (BMSCs) that are more inclined to differentiate into adipocytes rather than osteoblasts [4–6]. BMSCs lineage specification via transcriptional control is strictly orchestrated by key molecular signals to maintain bone-fat balance [7–9]. For example, RUNX2 and ALPL serve as crucial transcription factors (TFs) to initiate the osteoblastic lineage, while PPARγ and PGC1-α are master regulators of adipogenic differentiation. However, this does not explain the inverse relationship between osteoblastic lineage commitment accompanied by a coordinated inhibition of adipogenesis [10–12] or the decision of ultimate differentiation fates [10, 13, 14]. Thus, it is essential to explore the potential molecular switches that govern BMSCs differentiation fate during skeletal aging.

Accumulating evidence demonstrates that the regulation of mitochondrial dynamics and function is essential for BMSCs differentiation determination and cellular senescence [15–18]. Mitochondria are highly dynamic organelles that undergo cyclic fission-fusion and fragmentation-elongation and determine BMSCs differentiation fates by regulating oxidative phosphorylation (OxPhos) and energy metabolism [19, 20]. Aberrant fission and/or fusion leads to mitochondrial dysfunction, and in turn pathological BMSCs commitment and cellular senescence [16, 21–24]. Growing evidence supports the cross-regulatory role of RNA transcripts between nuclei and mitochondria [25]. Among the abundant transcripts in the nucleoplasmic compartment, termed paraspeckles, lncRNA nuclear-enriched abundant transcript 1 (NEAT1) retains mRNAs of nuclear-encoded mitochondrial proteins in response to mitochondrial stress [26]. Meanwhile, NEAT1, as transcribed by ATF2, regulates mitochondrial dynamics and function by influencing paraspeckle numbers and assembly [25]. Therefore, the specific molecular switches modulating BMSCs differentiation determination and cellular senescence by regulating mitochondrial function need to be determined.

Transcriptional regulation of BMSCs for self-renewal and differentiation demands precise coordination of key molecular switches and state-specific gene expression patterns [27–29]. Pluripotency-related genes, including SOX2, OCT4, and NANOG, serve as core TFs for self-renewal maintenance by promoting the transcription of specific genes, whose downregulation also leads to irreversible cellular senescence as it fails to maintain genomic stability [30]. Importantly, in response to the induction of BMSCs differentiation, lineage commitment-associated molecular switches and transcription networks depend on multi-spatiotemporal epigenetic responses of promoter and enhancer regions. A previous study reported that NEAT1 is transcriptionally controlled by super-enhancers (SEs) [31]. As large domains of clustered enhancers, SEs regulate cell-type specific genes and have been linked to control and definition of cell identity by amassing rapid transcriptional effectors and mediating high efficiency of transcriptional machinery [32–34]. Thus, we wanted to explore whether SEs and related molecular switches are involved in lineage commitment and self-renewal during BMSCs aging.

Here, we profiled transcriptional and epigenetic regulation differences between adipogenesis and osteogenesis and identified the SEs-associated lncRNA NEAT1 as a pivotal molecular switch in BMSCs differentiation. We confirmed NEAT1 as a miRNA sponge that significantly influences mitochondrial function, adipogenesis and osteogenesis and as a protein-binding scaffold connecting SOX2 and CDK2 and then deactivating core pluripotency TFs. NEAT1 promoted CSF1 secretion by BMSCs and strengthened osteoclast differentiation. Neat1 small interfering RNA (si-Neat1) delivery effectively prevented age-related bone loss. To our knowledge, this is the first evidence linking mitochondrial stresses to pluripotency maintenance through lncRNA NEAT1, contributing to the knowledge regarding lineage determination of BMSCs during skeletal aging.

## Results

### NEAT1 is an abundant marker gene during BMSCs differentiation and senescence

**Fig. 1:**
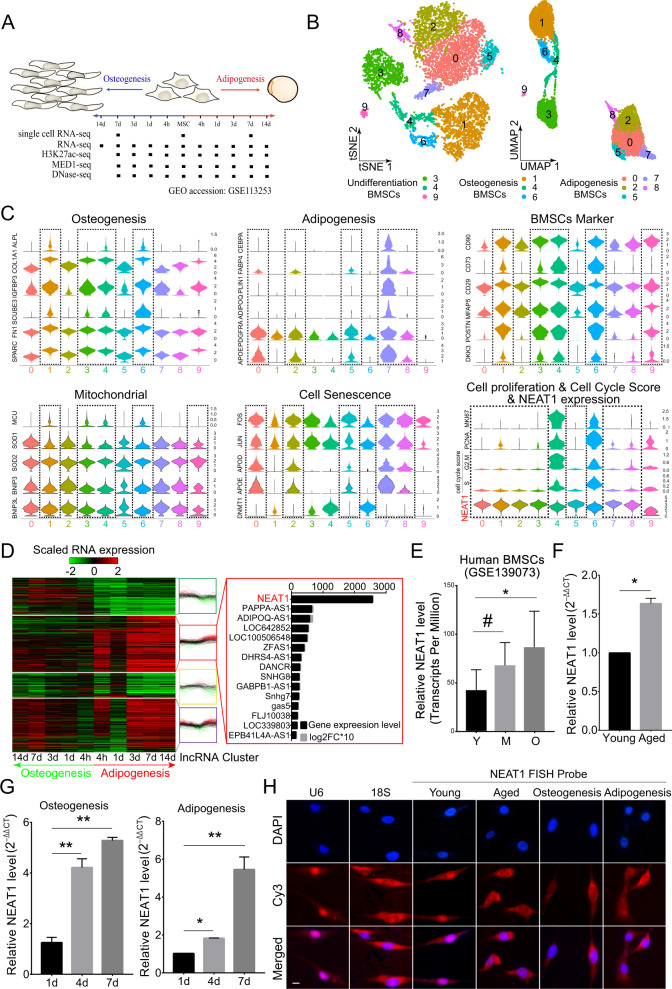
NEAT1 is an abundant marker gene during BMSCs differentiation and senescence. **A** Overview of human BMSCs osteogenic and adipogenic differentiation and time points for harvesting single-cell RNA-seq, RNA-seq, H3K27ac-seq, Med1-seq, and DNase-seq samples. **B** T-distributed stochastic neighbor embedding (t-SNE) and uniform manifold approximation and projection (UMAP) visualizations of all cell clusters identified using the computational pipeline. **C** Violin plots of related marker genes from ten clusters. NEAT1 is marked in red. **D** Heat map of K-means clustering of 1090 differentially expressed lncRNAs (log_2_FC > 1.0, *p* value <0.01) during BMSCs differentiation. NEAT1 is marked in red. **E** The expression levels (transcripts per million) of NEAT1 in BMSCs derived from subjects of different ages (Y: young, M: middle-aged, O: aged, *n* = 5). **F** qRT-PCR analysis of NEAT1 expression in BMSCs derived from young and aged subjects (*n* = 3). **G** qRT-PCR analysis of NEAT1 expression in BMSCs during osteogenic and adipogenic differentiation. **H** FISH localization of NEAT1 in BMSCs during cell senescence and differentiation. U6 and 18S rRNA were used as positive controls for the nuclear and cytoplasmic fractions, respectively. Scale bar: 20 μm. The results were presented as means ± S.D. *p < 0.05; **p < 0.01; ^#^*p* > 0.05 by Student’s *t* test and one-way ANOVA.

To explore the fate switches involved in the adipogenic-osteogenic imbalance in aged BMSCs, we profiled transcriptome and epigenomics differences between osteogenesis and adipogenesis by analyzing single-cell RNA-seq, RNA-seq, DNase-seq, Med1-seq, and H3k27ac-seq of human BMSCs (Fig. 1A). Dimensional reduction analysis using t-distributed stochastic neighbor embedding (t-SNE) and uniform manifold approximation and projection revealed a diversity of undifferentiated and differentiated BMSCs cell types (Fig. 1B). We mapped the differentiation trajectories of undifferentiated BMSCs, osteogenic BMSCs, and adipogenic BMSCs (Fig. S1A). BMSCs clustering and biforked trajectories indicated that adipogenic differentiation involved isolating changes in gene expression compared to osteogenic differentiation. Consistent with a previous study [35], at the transcriptome level, undifferentiated BMSCs were closer to osteoblasts than to adipocytes. In total, 1468 marker genes from ten clusters displayed a dynamic expression pattern (Supplementary Table 1). Among them, more terminal clusters exhibited differential gene expression levels of BMSCs markers, mitochondrial function, cellular senescence, and cell cycle-related genes, in which lncRNA NEAT1 demonstrated significant and specific high expression levels (Figs. 1C, S1B). Importantly, compared to undifferentiated clusters, all terminal differentiation clusters manifested significant upregulation of NEAT1 and were accompanied by cell marker loss, cell cycle arrest, and cellular senescence (Figs. 1C, S1D). Pseudotime analysis of RNA-seq also supported these findings, and the NEAT1’ shorter transcript NEAT1_1 (NR_028272) was the most abundant marker RNA during BMSCs adipogenesis (Figs. 1D, S1C). Meanwhile, we validated the upregulation of NEAT1 coupled with fat metabolism and cell cycle regulation (Fig. S1E). Additionally, NEAT1 but not NEAT1_2 exhibited age- and terminal differentiation-associated upregulation in human and mouse BMSCs (Figs. 1E–G, S1F–H). qRT-PCR of the nucleus and cytoplasm and FISH assays further demonstrated the transposition of NEAT1 from the nucleus to the cytoplasm during BMSCs senescence and differentiation (Figs. 1H, S1I–M). Importantly, NEAT1 was simultaneously upregulated in the nucleus and cytoplasm in aged BMSCs (Fig. S1K–L).

Taken together, these findings reveal that osteogenesis involves the induction of many genes that are already active in undifferentiated BMSCs, whereas adipogenesis is characterized by BMSCs markers loss and NEAT1_1 upregulation. Therefore, NEAT1_1 may be involved in the adipogenic lineage fate of aged BMSCs.

### SE-associated NEAT1 is activated by ATF2

**Fig. 2:**
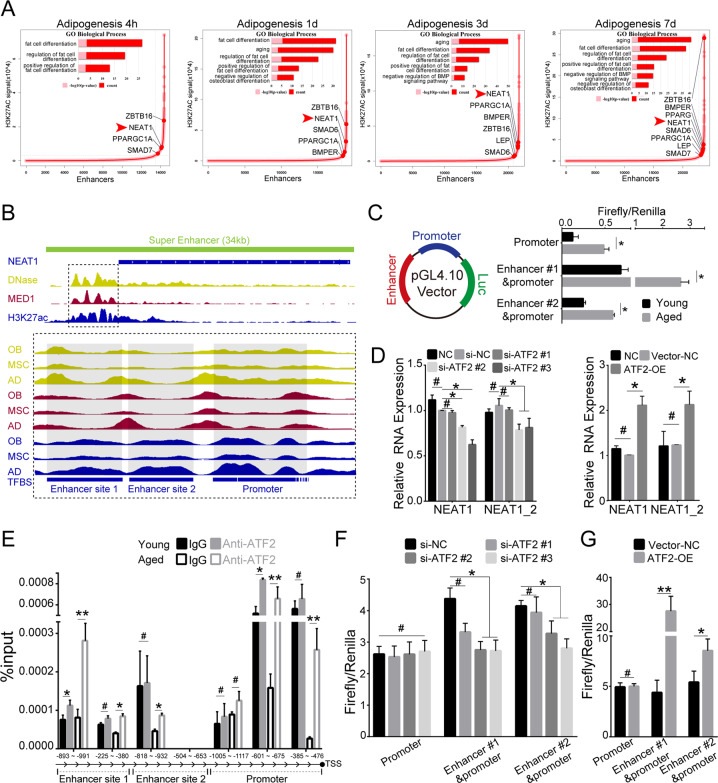
SE-associated NEAT1 is activated by ATF2. **A** Hockey stick plots of the rank order of H3K27ac signals for all enhancers in BMSCs during adipogenic differentiation. Inserted panels of selected GO functional categories of SE-associated genes. The red arrow indicates the NEAT1-related SE. **B** Integrative Genomics Viewer (IGV) of H3K27ac-seq with DNase-seq and Med1-seq read density in NEAT1 of undifferentiated BMSCs and BMSCs with multilineages differentiation (OB osteogenic differentiation, AD adipogenic differentiation). **C** Promoter/enhancer reporter assays in young/aged BMSCs validated the age-related promoter/enhancer function of NEAT1. **D** NEAT1 expression level of BMSCs transfected with normal control or si-ATF2 (left panel) and control vector or ATF2 plasmid (right panel). **E** ChIP assays showed ATF2 binding sites in the NEAT1 promoter/enhancer region of young/aged BMSCs. **F**, **G** ATF2 knockdown and overexpression showed a significant influence on NEAT1 promoter/enhancer region activity. The results were presented as means ± S.D. *p < 0.05; **p < 0.01; ^#^*p* > 0.05 by Student’s *t* test and one-way ANOVA.

Recent work has demonstrated that SE-associated genes are particularly involved in cell development [36, 37], and differentiation [38]. Considering the upregulation of NEAT1 in the terminal differentiation of BMSCs, we investigated the upstream epigenomic regulation of NEAT1. H3K27ac-seq data generated in undifferentiated and differentiated BMSCs were analyzed for the identification of SEs (Figs. 2A, S2A). The results showed that linage differentiation-related marker genes, such as ALPL and PPARG, performed SEs enrichment (Figs. 2A, S2A). Notably, unlike osteogenesis, adipogenesis specifically affected the biological process of cellular senescence and negatively regulated osteogenesis, as shown by gene ontology enrichment analysis of SE-associated genes, in which NEAT1 was associated with SE (Fig. 2A). The differentiation-specific active nature of this SE was corroborated by the co-occupancy of both DNase and Med1 (Figs. 2B, S2B). Considering the simultaneous transcriptomic and epigenomic activity of NEAT1 during BMSCs differentiation, we further identified that NEAT1 transcript upregulation accompanied epigenetic alterations in aged BMSCs (Fig. 2C). We also overlapped TF binding sites between the NEAT1 promoter and enhancer region using the JASPAR database. The majority of TF binding sites were simultaneously located in the promoter-enhancer region, including core TFs of pluripotency maintenance (SOX2, OCT4), linage differentiation (RUNX2, CEBPB), cellular senescence (FOS, JUN), and mitochondrial function (ATF2) (Fig. S2C, Supplementary Table 2). Genome-wide RNAi screenings of NEAT1 have identified ATF2 as a potential transcriptional regulator involved in mitochondrial function [25]. Therefore, we investigated whether ATF2 mediated the expression of NEAT1 in BMSCs fate determination and found that ATF2 knockdown or overexpression resulted in corresponding changes in NEAT1 levels (Figs. 2D, S2D). ChIP assays further demonstrated that ATF2 bound to the promoter-enhancer region of NEAT1, and the binding activity was substantially increased in aged BMSCs (Fig. 2E). Meanwhile, ATF2 knockdown or overexpression inhibited or promoted the promoter-enhancer activity of NEAT1, respectively (Fig. 2F, G). These results indicate that SE-associated NEAT1 is transcriptionally activated by mitochondrial stress factor ATF2 during BMSCs aging and adipogenesis.

### NEAT1 regulates the lineage fates of BMSCs by impairing mitochondrial function

**Fig. 3:**
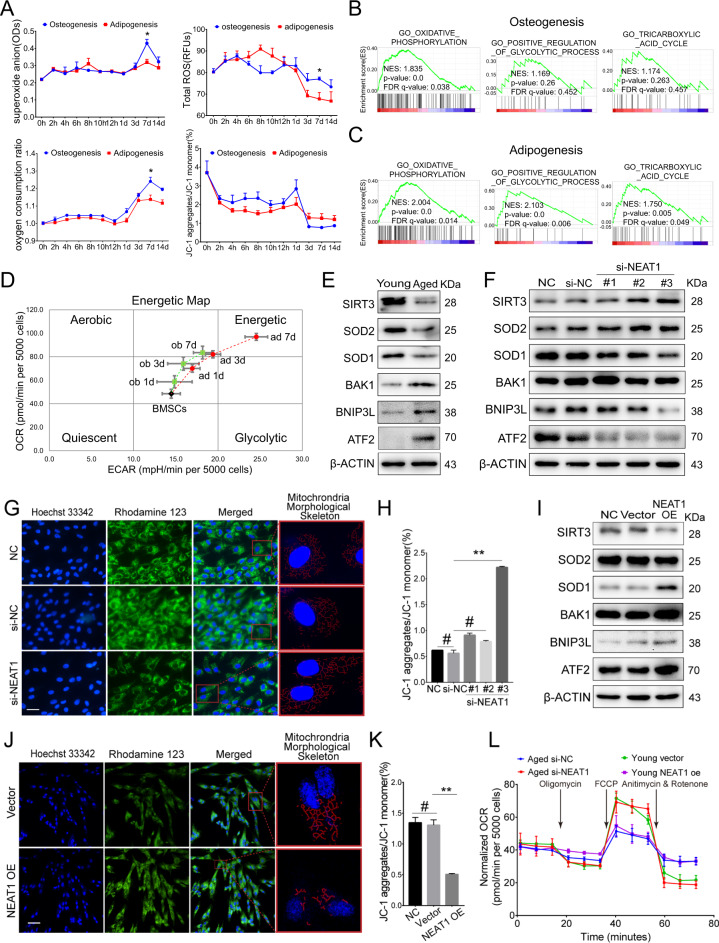
NEAT1 regulates the lineage fates of BMSCs by impairing mitochondrial function. **A** Assessment of superoxide anion, reactive oxygen species, oxygen consumption, and MMP during BMSCs osteogenic and adipogenic differentiation. **B** Gene set enrichment analysis of energy metabolism-related pathways during BMSCs osteogenic differentiation and (**C**) adipogenic differentiation. **D** Energy map based on the ATP rate (OCAR/ECAR) of the energy metabolic pathway of BMSCs osteogenesis and adipogenesis. **E** Western blotting showed decreased mitochondrial function in aged BMSCs. **F** si-NEAT1 decreased the expression levels of mitochondrial membrane related ATF2, BAK1, BNIP3L, while improved the SIRT3/SOD2 pathway of aged BMSCs during osteogenic differentiation. **G** si-NEAT1 in aged BMSCs during osteogenic differentiation resulted in MMP and mitochondrial morphological skeleton recovery. **H** The MMP assay kit with JC-1 showed that si-NEAT1 improved MMP. **I** Western blotting showed that NEAT1 overexpression increased the expression levels of mitochondrial membrane related ATF2, BAK1, BNIP3L, while suppressed the SIRT3/SOD2 pathway of young BMSCs during osteogenic differentiation. **J** NEAT1 overexpression in young BMSCs during osteogenic differentiation led to a decline in MMP and mitochondrial morphological skeleton fragmentation. **K** The JC-1 assay exhibited decreased MMP in young BMSCs with NEAT1 overexpression. **L** Bioenergetic profiling of the BMSCs osteogenesis process with si-NEAT1 and NEAT1 overexpression approaches in a Mito stress test measuring mitochondrial respiration. Scale bars: 50 μm (**G**); 100 μm (**J**). The results were presented as means ± S.D. *p < 0.05; **p < 0.01; ^#^*p* > 0.05 by Student’s *t* test and one-way ANOVA.

To investigate whether NEAT1-mediated BMSCs fates are involved in mitochondrial function, we induced osteogenic and adipogenic differentiation of BMSCs and subsequently analyzed mitochondrial function and energy metabolism signatures. Distinguished from the adipogenic process, the osteogenic process required higher mitochondrial quality, including mitochondrial metabolites (superoxide anion, reactive oxygen species), oxygen consumption, and mitochondrial membrane potential (MMP) (Fig. 3A). Notably, the difference in mitochondrial quality between the osteogenic and adipogenic differentiation processes was accompanied by NEAT1 upregulation at a key time point on day 7 (Figs. 3A, 1D, G). Gene set enrichment analysis showed that osteogenesis solely depended on OxPhos (Fig. 3B), while adipogenesis had multiple energy metabolic pathways (Fig. 3C). The energetic map based on the ATP rate (OCAR/ECAR) also revealed a relatively single energy metabolic pathway of OxPhos during osteogenic differentiation of BMSCs (Fig. 3D). Importantly, aged BMSCs exhibited poor mitochondrial function reflected by inhibition of the SIRT3/SOD2 pathway and impaired MMP (Figs. 3E, S3A).

We next implemented NEAT1 small interfering RNA (si-NEAT1) in aged BMSCs (Fig. S3B) and found that mitochondrial quality and SIRT3/SOD2 pathway were significantly improved (Fig. 3F), including MMP recovery and morphological network defragmentation (Figs. 3G, H, S3C). In contrast, NEAT1 overexpression in young BMSCs (Fig. S3D) showed impaired mitochondrial function (Fig. 3I), including MMP decline and morphological network fragmentation (Figs. 3J, K, S3E). Additionally, the Mito stress test validated the negative regulation of OxPhos activity by NEAT1 (Fig. 3L), and individual mitochondria in the si-NEAT1 group were significantly enlarged compared to those of the si-NC group (Fig. S3F). Mechanistically, we confirmed that the mitochondrial function-related gene BNIP3L was regulated by NEAT1 (Fig. S3G, H). Altogether, our data indicate that NEAT1 regulates the lineage fates of BMSCs by impairing mitochondrial function.

### NEAT1 attenuates BMSCs pluripotency by scaffolding SOX2 and CDK2

**Fig. 4:**
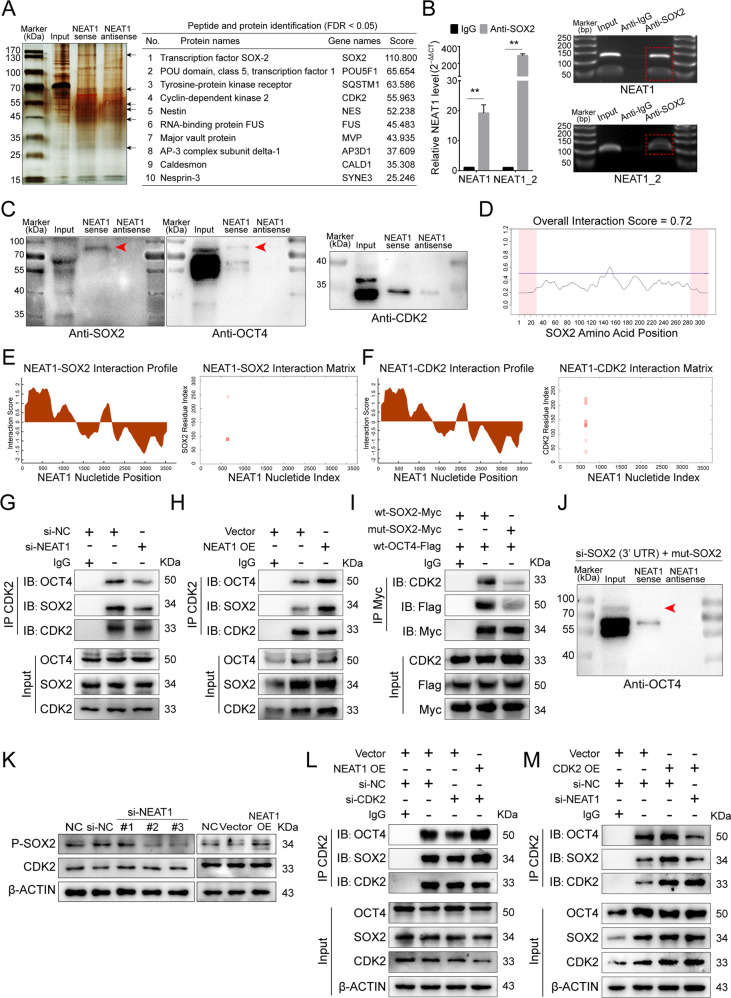
NEAT1 attenuates BMSCs pluripotency by scaffolding SOX2 and CDK2. **A** Silver staining of NEAT1 pulldown in undifferentiated BMSCs, left panel. Arrows show different bands between the sense and antisense lanes. List of the top ten differentially expressed proteins identified by mass spectrometry, FDR < 0.05, right panel (*n* = 3). **B** Expression levels of NEAT1 detected by qRT-PCR after RIP for SOX2 in BMSCs. **C** Western blotting showed NEAT1 pulldown of the SOX2/OCT4 complex and CDK2. The red arrow indicates the SOX2/OCT4 complex. **D** CatRAPID signature module prediction of the RNA-binding propensity for SOX2 protein followed by the prediction of RNA-binding regions. Overall interaction scores above 50% indicate the binding propensity. **E** CatRAPID fragment module prediction of the interaction profile and matrix between SOX2 and NEAT1. **F** Interaction between CDK2 and NEAT1. **G**, **H** Immunoprecipitation of SOX2 and OCT4 using an anti-CDK2 antibody. SOX2 and OCT4 were reduced in BMSCs transfected with si-NEAT1. By contrast, SOX2 and OCT4 expression increased in BMSCs overexpressing NEAT1. **I** Immunoprec**i**pitation of CDK2 and Flag-OCT4 using an anti-Myc antibody. CDK2 and Flag-OCT4 were reduced in 293T cells transfected with the mut-SOX2 (Ser249/250 mutation) plasmid. **J** Western blotting showed NEAT1 pulldown of BMSCs transfected with SOX2 small interfering and mut-SOX2 plasmid. **K** si-NEAT1 and NEAT1 overexpression downregulated and upregulated ser249/250-phosphorylated SOX2, but not CDK2, respectively. **l** Interaction among CDK2, SOX2, and OCT4 was identified in BMSCs co-transfected with si-CDK2 and NEAT1 plasmid by coimmunoprecipitation. **M** The interaction among CDK2, SOX2, and OCT4 was confirmed in BMSCs co-transfected with the CDK2 plasmid and si-NEAT1 by coimmunoprecipitation. The results were presented as means ± S.D. **p < 0.01 by Student’s *t* test and one-way ANOVA.

To explore NEAT1’s interaction with RNA-binding proteins, we designed a specific biotin-labeled NEAT1 probe to perform an RNA pulldown assay in BMSCs. The silver staining results revealed enrichment of several bands of proteins combined with NEAT1 (Fig. 4A left). Protein mass spectrometry analysis was used to identify differentially expressed proteins, and SOX2, OCT4, and CDK2 ranked forward in the recognized protein list (Fig. 4A right, Supplementary Table 3). The RIP assay revealed that antibodies against SOX2, OCT4, and CDK2 pulled down abundant NEAT1 compared to IgG (Figs. 4B, S4A, B). We further identified NEAT1 bound to the SOX2/OCT4 complex (Fig. 4C). Importantly, pluripotent maintenance factors acts as key regulator in cell cycle control and cellular senescence remission, while the SOX2/OCT4 protein complex can dominate and synergize with NANOG in maintaining pluripotency and self-renewal of adult stem cells [39]. Therefore, we investigated whether potential binding sites existed between NEAT1 and SOX2/OCT4, and the results of the catRAPID server demonstrated NEAT1-SOX2 and NEAT1-OCT4 interaction scores of 0.72 and 0.68, respectively (Figs. 4D, S4C). Next, catRAPID fragments further revealed that NEAT1 simultaneously bound to SOX2, OCT4, and CDK2 at similar nucleotide positions with high propensities (Figs. 4E, F, S4D). Multiple labeling of SOX2, OCT4, and CDK2 immunofluorescence and NEAT1 FISH further confirmed the colocalization of NEAT1 and SOX2/OCT4/CDK2 during BMSCs aging (Fig. S4E, F). Notably, the predicted interaction domain of SOX2 contains several identified consecutive serine phosphorylation sites: Ser249, Ser250, and Ser251 (Fig. 4E). A previous study verified that Cdk2 interacts with Sox2 and phosphorylates Sox2 at Ser253 (human SOX2 Ser251) [40]. Our results further revealed that NEAT1 knockdown attenuated CDK2-SOX2 interactions, while NEAT1 overexpression strengthened the interactions (Fig. 4G, H).

Considering that SOX2 is often a compound complexed with OCT4 in pluripotency maintenance [41–43], we further verified that SOX2 with the Ser249/250 phosphorylation mutation resulted in weakened SOX2/OCT4 complexes and SOX2-CDK2 interactions (Figs. 4I, S4G). Furthermore, we knocked down endogenous SOX2 using small interfering RNA of the SOX2 3′UTR and transferred it to the SOX2 plasmid with the Ser249/250 phosphorylation mutation (Fig. S4H). Then, an RNA pulldown assay showed that interactions between NEAT1 and the SOX2/OCT4 complex was not detected (Fig. 4J). Meanwhile, we showed that NEAT1 could not bind with Ser249/250 phosphorylation-mutated SOX2 (Fig. S4I). NEAT1 knockdown and overexpression resulted in downregulation and upregulation of phosphorylation SOX2 (Ser249/250), respectively, while expression levels of CDK2 did not change (Fig. 4K). Additionally, as a scaffold, NEAT1 overexpression rescued CDK2-SOX2 interactions, which were suppressed by si-CDK2 transfection (Figs. 4L, S4J), and ascending CDK2-SOX2 interactions activated by CDK2 overexpression were damaged by NEAT1 knockdown (Figs. 4M, S4K). Compared to young BMSCs, aged BMSCs exhibited cycle arrest and cellular senescence (Fig. S4L–M). NEAT1 knockdown in aged BMSCs resulted in G0/G1 cycle arrest release and senescence remission (Fig. S4N, O), while NEAT1 overexpression induced the opposite effect (Fig. S4P, Q). Altogether, our data demonstrate that NEAT1 attenuates BMSCs pluripotency by scaffolding the RNA-binding proteins SOX2 and CDK2 and deactivating core pluripotent TFs.

### NEAT1 improves adipogenesis and attenuates osteogenesis in aged BMSCs

**Fig. 5:**
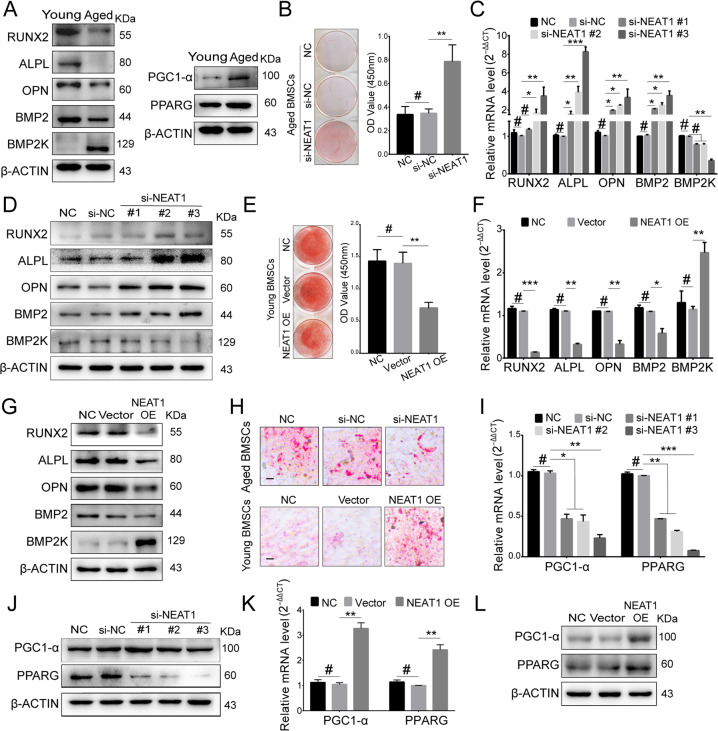
NEAT1 improves adipogenesis and attenuates osteogenesis in aged BMSCs. **A** Western blotting revealed that osteogenesis was significantly attenuated in aged BMSCs, while adipogenesis was markedly strengthened. **B–D** Alizarin red staining, qRT-PCR, and western blotting showed that si-NEAT1 rescued the osteogenesis decline in aged BMSCs. **E–G** Alizarin red staining, qRT-PCR, and western blotting showed that NEAT1 overexpression decreased osteogenesis in young BMSCs. **H**–**L** Oil red O staining, qRT-PCR, and western blotting showed that NEAT1 promoted adipogenesis. Scale bar: 200 μm. The results were presented as means ± S.D. *p < 0.05; **p < 0.01; ***p < 0.001; ^#^*p* > 0.05 by Student’s *t* test and one-way ANOVA.

Considering the upregulation of NEAT1 in aged BMSCs and its effects on fates determination, we next explored the roles of NEAT1 in aged BMSCs differentiation. Aged BMSCs exhibited a decreased osteogenic capacity and an increased adipogenic tendency (Fig. 5A). During osteogenic differentiation of aged BMSCs, NEAT1 knockdown markedly improved osteogenesis of BMSCs (Figs. 5B–D, S5A). In contrast, NEAT1 overexpression in young BMSCs decreased osteogenesis of BMSCs (Figs. 5E–G, S5B). Additionally, loss- and gain-of-function of NEAT1 caused decreased and increased adipogenic differentiation of BMSCs, respectively (Fig. 5H–L). Notably, accompanied by improved adipogenesis, NEAT1 overexpression promoted expression levels of BMP2K, a protein kinase with a regulatory role in attenuating osteoblast differentiation. These data identify NEAT1 as a regulator of lineage differentiation in BMSCs.

### NEAT1 promotes paracrine CSF1-dependent osteoclastic activation

**Fig. 6:**
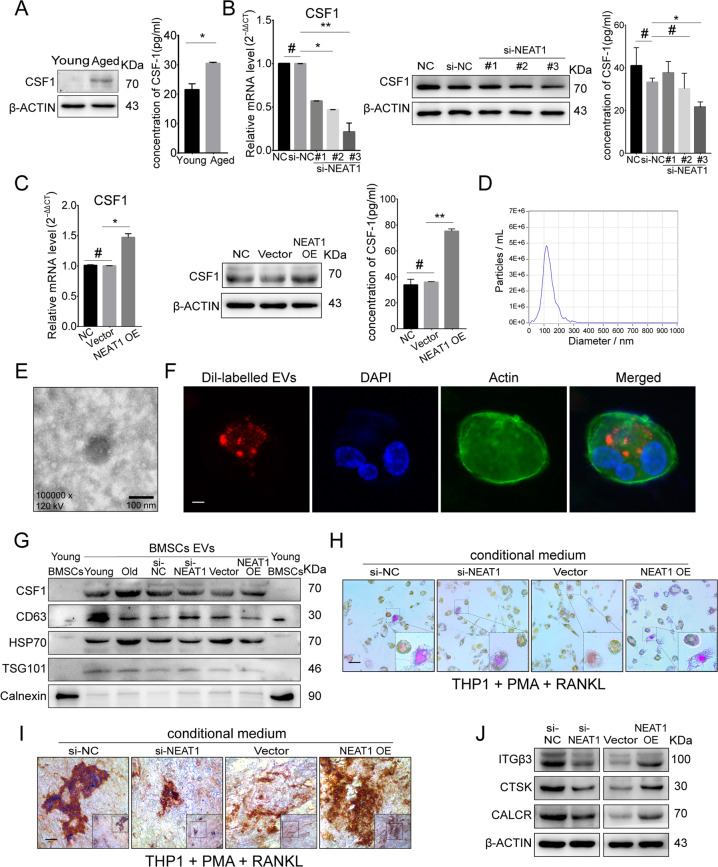
NEAT1 promotes paracrine CSF1-dependent osteoclastic activation. **A** Western blotting and ELISA revealed upregulated CSF1 expression and secretion in aged BMSCs. **B**, **C** qRT-PCR, Western blotting, and ELISA showed that NEAT1 enhanced CSF1 expression and secretion. **D** NTA analysis determined the size distribution of the isolated extracellular vesicles (EVs). **E** EVs were visualized on a transmission electron microscope (TEM). **F** DiI-labeled EVs from BMSCs incorporated into THP-1 cells were visualized by fluorescence microscopy. **G** Western blotting of CSF1, CD63, HSP70, TSG101, and Calnexin was performed on EVs and BMSCs. **H–J** EVs derived from BMSCs with si-NEAT1 and NEAT1 overexpression influenced THP-1 differentiation, as detected by TRAP staining, the bone resorption assay, and western blotting. Scale bars: 100 nm (**E**); 20 μm (**F**); 100 μm (**H**, **I**). The results were presented as means ± S.D. *p < 0.05; **p < 0.01; ^#^*p* > 0.05 by Student’s *t* test and one-way ANOVA.

BMSCs senescence is commonly considered as an initial representation of bone degeneration [44, 45], involving wide secretion of cytokines to other cells via the paracrine pathway and extracellular vesicles (EVs) contributing to aging-associated tissue dysfunction [46, 47]. CSF1, a secretion protein, was markedly increased in aged BMSCs, as well as in the cellular supernatant (Figs. 6A, S6A). NEAT1 knockdown resulted in downregulated CSF1 levels in cells and supernatant (Fig. 6B), while NEAT1 overexpression displayed the opposite results (Fig. 6C). To investigate EVs secreted from BMSCs, we purified EVs by super-centrifugation, and nanoparticle-tracking analysis of EVs demonstrated the size distribution (Fig. 6D). We observed EVs via TEM, and confirmed the incorporation into THP-1 cells using DiI labeling (1,1′-dioctadecyl-3,3,3′,3′-tetramethylindocarbocyanine perchlorate) (Fig. 6E, F). High expression levels of CD63, HSP70, and TSG101 were detected in these EVs, while Calnexin was barely detected (Fig. 6G). Importantly, EVs derived from BMSCs contained high levels of CSF1, which was positively associated with NEAT1 levels (Fig. 6G). Next, for induction of osteoclasts, THP-1 cells were cocultured with supernatant from aged BMSCs, aged BMSCs with NEAT1 knockdown, young BMSCs, or young BMSCs with NEAT1 overexpression, in which multinucleated osteoclasts with high activity of tartrate-resistant acid phosphatase (TRAP) were observed in groups of aged BMSCs and young BMSCs with NEAT1 overexpression (Figs. 6H, S6B). Meanwhile, THP-1 cells treated with the supernatant from aged BMSCs with NEAT1 knockdown and young BMSCs demonstrated decreased TRAP activity (Figs. 6H, S6B). Furthermore, decreased bone resorption activity was observed in bovine bone slides cultured with supernatant from si-NEAT1-transfected BMSCs (Figs. 6I, S6C), while increased bone resorption pits were observed in bovine bone slides treated with supernatant from NEAT1 plasmid-transfected BMSCs (Figs. 6I, S6C). In line with these findings, the osteoclast activity-related factors ITGβ3, CTSK, and CALCR showed corresponding changes (Fig. 6J). Thus, for extracellular communication, NEAT1 promotes CSF1 secretion by aged BMSCs via EVs delivery and strengthens osteoclastic differentiation (Fig. S6D).

### NEAT1 serves as a miR-27b-3p sponge and mediates the characteristics of aged BMSCs

**Fig. 7:**
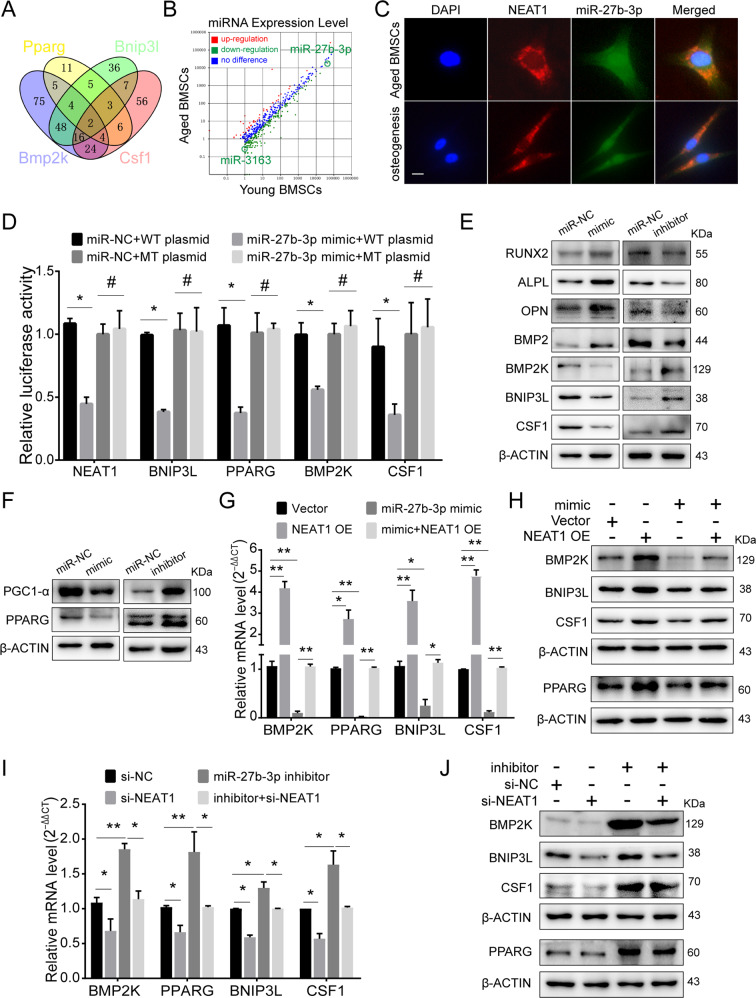
NEAT1 serves as a miR-27b-3p sponge and mediates the characteristics of aged BMSCs. **A** Venn diagram showing four miR-27b-3p- and miR-3163-targeted genes predicted by TargetScan. **B** miRNA profiling in young and aged BMSCs showed downregulated miR-27b-3p and miR-3163 in aged BMSCs. **C** Colocalization of NEAT1 and miR-27b-3p was detected by FISH in aged BMSCs and BMSCs osteogenic differentiation. **D** Luciferase reporters of NEAT1, BNIP3L, PPARG, BMP2K, and CSF1 and luciferase activity in 293T cells co-transfected with miR-27b-3p mimic. **E**, **F** Western blotting showed the expression of BNIP3L, PPARG, BMP2K, and CSF1 following exposure to the miR-27b-3p mimic and inhibitor. **G**, **H** qRT-PCR and Western blotting revealed that the miR-27b-3p mimic decreased the upregulation of BNIP3L, PPARG, BMP2K, and CSF1 by NEAT1 overexpression. **I**, **J** qRT-PCR and Western blotting showed thar the miR-27b-3p inhibitor rescued the downregulation of BNIP3L, PPARG, BMP2K, and CSF1 by si-NEAT1. Scale bar: 20 μm. The results were presented as means ± S.D. *p < 0.05; **p < 0.01; ^#^*p* > 0.05 by Student’s *t* test and one-way ANOVA.

Given that lncRNAs have been widely explored as miRNA sponges and NEAT1 is abundant in the cytoplasm of aged BMSCs, we speculated that NEAT1 might act as a miRNA sponge to regulate phenotypic genes. The NEAT1-related regulatory genes BNIP3L, PPARG, BMP2K, and CSF1 were used to predict potential microRNAs by TargetScan (Supplementary Table 4). By overlapping four groups of potential miRNAs, two candidate miRNAs, miR-27b-3p and miR-2763, were screened out (Fig. 7A). According to microRNA microarrays between young BMSCs and aged BMSCs, we further detected high expression levels and significant downregulation of miR-27b-3p in aged and differentiated BMSCs, respectively (Figs. 7B, S7A). miR-27b-3p downregulation in aged BMSCs was reversed by NEAT1 knockdown (Fig. S7B), while NEAT1 overexpression in young BMSCs resulted in miR-27b-3p downregulation (Fig. S7C). FISH assays confirmed that NEAT1 and miR-27b-3p were colocalized in the cytoplasm (Fig. 7C), and NEAT1 pulldown identified an interaction between NEAT1 and miR-27b-3p (Fig. S7D). Meanwhile, biotin-labeled miR-27b-3p pulldown demonstrated the interaction of miR-27b-3p with NEAT1 and target genes (Fig. S7E). To further verify their direct interaction, we constructed dual-luciferase reporters and found that the miR-27b-3p mimic reduced the luciferase activity of reporter vectors containing NEAT1, BNIP3L, PPARG, BMP2K, and CSF1 relative to NC treatment (Fig. 7D). By AGO2 immunoprecipitation and NEAT1 pulldown assays, we verified the binding of AGO2 and NEAT1, indicating that NEAT1 acts as a miRNA sponge (Fig. S7F, G).

To investigate the function of miR-27b-3p, we employed loss- and gain-of-function miR-27b-3p and found corresponding changes in BNIP3L, PPARG, BMP2K, and CSF1 (Fig. 7E, F). To confirm the effect of the NEAT1-miR-27b-3p interaction on target genes, we co-transfected miR-27b-3p inhibitor and si-NEAT1, as well as miR-27b-3p mimic and NEAT1 plasmid. qRT-PCR and western blot assays demonstrated that NEAT1 overexpression significantly increased the mRNA and protein levels of target genes, whereas the miR-27b-3p mimic decreased the above markers (Fig. 7G, H) and induced osteogenesis markers (Fig. S7H). Similarly, knockdown of NEAT1 suppressed the expression levels of target genes, while the miR-27b-3p inhibitor rescued the downregulation of target genes and attenuated osteogenesis (Figs. 7I, J, S7I). These results suggest that NEAT1 regulates mitochondrial function, lineage differentiation, and CSF1 secretion in aged BMSCs by sponging miR-27b-3p.

### si-Neat1 delivery prevents bone loss and marrow fat accumulation in aged mice

**Fig. 8:**
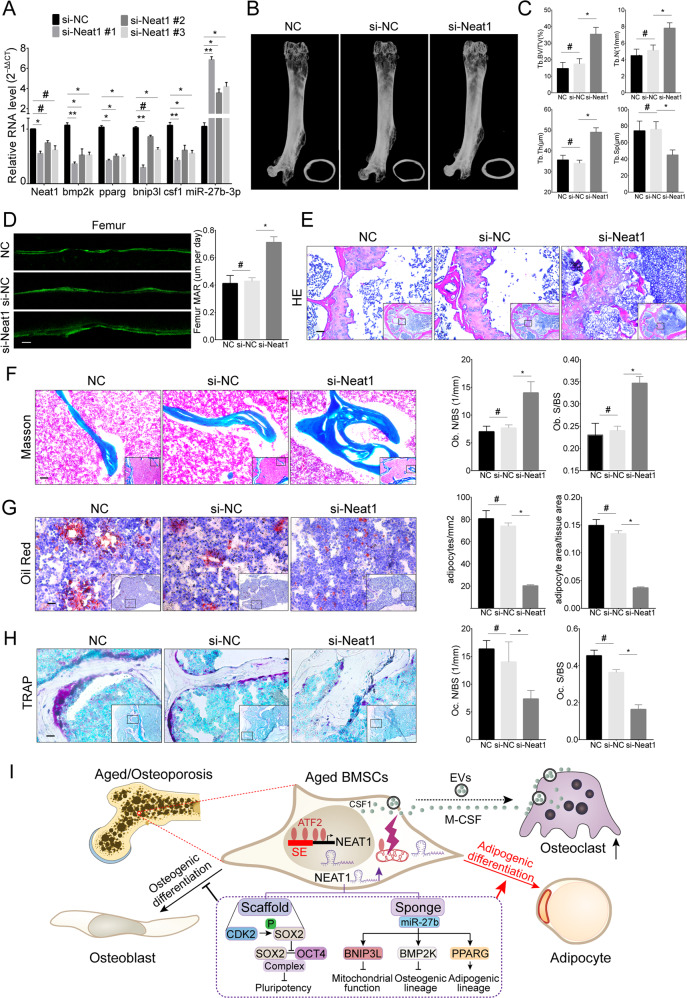
si-Neat1 delivery prevents bone loss and marrow fat accumulation in aged mice. **A** qRT-PCR analysis of the expression levels of Neat1 and related genes in mouse BMSCs transfected with si-Neat1. **B**, **C** Micro-CT images and quantitative CT analysis were performed in the distal femur from 18-month-old mice treated with control, si-NC and si-Neat1 (*n* = 5). **D** Representative images of dynamic histomorphometry of trabecular bone with quantification of the mineralization apposition rate (MAR). **E**, **F** Representative images of HE and Masson’s trichrome staining showed that bone structure and osteoblasts were significantly increased in aged mice treated with si-Neat1. **G** Oil red O staining of the distal femur and quantification of adipocytes indicated that adipogenesis was markedly decreased in aged mice treated with si-Neat1. **H** TRAP staining of the distal femur and quantification of OC.N/B.Pm (osteoclast number per bone perimeter) and OC.N/BS (osteoclast number per bone surface) indicated that the osteoclast numbers were significantly decreased in aged mice with si-Neat1 treatment. **I** A model by which NEAT1 regulates the lineage fates of BMSCs in the aged bone marrow microenvironment. Scale bars: 20 μm (**D**); 200 μm (**E**); 100 μm (**F**); 50 μm (**G**, **H**). The results were presented as means ± S.D. *p < 0.05; **p < 0.01; ^#^*p* > 0.05 by Student’s *t* test and one-way ANOVA.

NEAT1 is a human-mouse homologous gene with 95% sequence overlap. We first confirmed a significant increase of Neat1 in aged mice BMSCs (Fig. S1G). The corresponding expression changes of Neat1 and above target genes were verified using si-Neat1 (Fig. 8A). Next, chemically modified si-Neat1 was delivered into aged mice (18 months old) via the caudal vein once a week (Fig. S8A), and bioluminescence was captured using an IVIS Spectrum Xenogen Imaging System (Caliper Life Sciences) for detecting si-Neat1 distribution and maintenance (Fig. S8B). After 6 weeks of administration, important organs were collected for observation (Fig. S8C). qRT-PCR was used to detect the expression of Neat1, bmp2k, pparg, bnip3l, csf1, and miR-27b-3p in BMSCs of young mice (si-NC injection), aged mice (si-NC injection), and si-NEAT1-treated aged mice, and the results showed NEAT1 downregulation, miR-27b-3p upregulation, and target genes downregulation in aged mice treated with si-NEAT1 (Fig. S8D). Furthermore, double immunofluorescence labeling of Nestin (a BMSCs marker) and Neat1 FISH directly revealed that si-NEAT1 treatment targeted BMSCs (Fig. S8E). Meanwhile, compared to the control group, the si-Neat1 group demonstrated significant improvement in bone mass and cortical bone thickness (Fig. 8B). Bone quality enhancement was also reflected in trabecular bone morphological analysis, including increased trabecular bone volume, thickness, and numbers as well as decreased trabecular bone separation (Fig. 8C). Dynamic histomorphometry revealed that the si-Neat1 group displayed elevated trabecular and endosteal bone formation rates (Fig. 8D). Notably, compared to the control group, si-Neat1 delivery enhanced trabecular bone structure and suppressed adipogenesis and osteoclastogenesis (Figs. 8E–H, S8F). In addition, no obvious pathological changes were observed in the lung, spleen, kidney, brain, heart, or liver (Fig. S8C, G). These results suggest that si-Neat1 delivery prevents imbalanced bone-fat switching and progressive bone loss in skeletal aging.

## Discussion

Bone aging includes dysregulation of the bone-fat balance with decreased bone mass and accumulated marrow fat [8, 9]. The imbalance in bone-fat derived from the disproportionality of adipocytes and osteoblasts is delicately controlled by the differentiation commitment of BMSCs [4]. Recent studies have reported several endocrinal signals of bone-fat switches that initiate imbalanced osteogenesis-adipogenesis in response to adiposity [48], hormones [49], and aging [50]. Here, we identified that the SE-associated lncRNA NEAT1 was highly expressed in aged BMSCs, which may link the aberrant differentiation fate of adipogenic activation and osteogenic suppression. At the epigenetic level, our results suggest that osteoblast-specific genes exhibit corresponding SE activity during osteogenic differentiation, but adipogenesis is accompanied by negative regulation of osteogenesis and cellular senescence-related SE activity. Furthermore, a previous study [4] supported our findings and demonstrated that disruption of adipogenesis protectively maintained bone homeostasis. Notably, BMSCs with NEAT1 overexpression tended to differentiate into adipocytes, which are also characterized by cellular senescence hallmarks, suggesting that SE-derived NEAT1 governs the adipogenic differentiation fate of aged BMSCs.

A recent study of aging cell fates reported that chromatin instability and mitochondrial decline represent different types of terminal states [51], while mitochondrial decline dominates the aging process [52]. Consistent with a recent study [35], we confirmed that adipogenic differentiation possessing similar characteristics to cellular senescence was relatively more terminal, while osteogenic differentiation possessing mitochondria-dependent energy metabolism was closer to the undifferentiated BMSCs state. As a mitochondrial protein that regulates mitochondrial permeability in response to genotoxic stress, ATF2 transcriptionally regulates cellular growth and development by nuclear localization [53–56]. Consistent with a previous study [25], we demonstrated the high binding activity of ATF2 in the NEAT1 promoter/enhancer region. Beyond shaping mitochondrial functions via paraspeckle formation [25], we further identified that NEAT1 controls mitochondrial function by regulating mitochondrial gene expression, and downregulating NEAT1 in aged BMSCs markedly rescued mitochondrial dysfunction. In addition, energy metabolism during osteogenic differentiation solely depended on OxPhos, while adipogenic differentiation exploited diverse energy metabolic pathways. Nevertheless, whether NEAT1 influences the glycolysis pathway and regulates adipogenesis related energy metabolism is worthy of further study.

The pluripotency properties of BMSCs include the potential for self-renewal and pluripotent differentiation [1, 29]. Our in vitro studies confirmed that NEAT1 intrinsically aggravated BMSCs senescence by promoting cell cycle arrest and pluripotency-related genes reduction. A previous study reported that NEAT1 scaffolds RNA-binding proteins in a stable manner [57]. Our findings revealed that NEAT1 scaffolding CDK2-SOX2 and SOX2 Ser249/250 phosphorylation impaired the stability of the SOX2/OCT4 complex. Importantly, the SOX2/OCT4 complex has shown a more sufficient role than either SOX2 or OCT4 alone in pluripotency maintenance [41–43, 58], so NEAT1 governs BMSCs pluripotency by scaffolding the RNA-binding proteins SOX2 and CDK2 and deactivating the SOX2/OCT4 complex.

Mounting evidence demonstrates that accumulation of the senescence-associated secretory phenotype in the aged bone marrow microenvironment is the main cause of osteoblast-osteoclast imbalance due to aberrant osteoimmune responses and BMSCs lineage commitment [44, 45]. Consistent with previous reports [47], our data further verified that CSF1 regulated by NEAT1 in aged BMSCs can be extracellularly delivered by a paracrine pathway to directly induce osteoclastic differentiation. Additionally, we found that high CSF1 levels in EVs from aged BMSCs were delivered into THP-1 cells. In line with a previous study [59], intracellular CSF1 can modulate osteoclast differentiation, but the underlying mechanisms need to be further investigated.

In conclusion, our study determined that the highly expressed lncRNA NEAT1 is activated by ATF2 in aged BMSCs as a bone-fat switch that shapes mitochondrial function, regulates pluripotency maintenance, and governs differentiation commitment in a miRNA sponge and protein-binding scaffold manner. Moreover, considering the effect of NEAT1 on upregulated osteoclastic and adipogenic differentiation and decreased osteogenic capability, the application of si-NEAT1 to the bone marrow microenvironment might represent a new approach for the treatment of age-associated osteoporosis (Fig. 8I).

## Materials and methods

### Experimental animals

Animal experimental procedures were approved by the Laboratory Animal Care and Use Committee at Nanjing Medical University (Approval No. IACUC-1905049). After the knockdown efficiency of si-Neat1 was verified by transfection of mouse BMSCs, si-Neat1 which chemically modified with 5Col/2OMe was synthesized by RiboBio (Guangzhou, China). Eighteen-month-old C57BL/6 mice were randomly divided into three groups. The sample size and inclusion criteria of each group was confirmed with adequate power based on the literature and our previous experience [60]. Two groups received either si-NC or si-Neat1 at a dose of 100 nM once per week by caudal vein injection. The other group of mice received a comparable volume of PBS. In the animal experiments, the investigator was blinded to the group allocation. After 6 weeks, the mice were euthanized and the femurs were harvested for micro-CT and histological analysis.

### In vitro cell culture and differentiation

For human jaw bone-derived BMSCs isolation, young (18–32 years old) and aged (62–79 years old) volunteers with informed consent were involved in the current study. The inclusion criteria and cell culture protocols were conducted as previously described [60], in which BMSCs were performed for the following experiment after three passages. Human BMSCs-related procedures were approved by The Ethical Committee Department at Affiliated Hospital of Stomatology of Nanjing Medical University (Approval No. PJ2018-047-001). Regarding mouse BMSCs isolation, the bone marrow was flushed with α-MEM using an 18-gauge sterile needle inserted into the medullary cavity. The following protocols were performed in accordance with human BMSCs culture. To induce osteogenic differentiation, BMSCs were cultured in complete medium supplemented with 10^−7^ M dexamethasone, 10 mM β-glycerophosphate, and 50 μM ascorbic acid. For adipogenic differentiation induction, BMSCs were cultured in complete medium containing 1 μM dexamethasone, 10 mg/l of insulin, 0.2 mM indomethacin, and 500 μM 3-isobutyl-1-methylxanthine (IBMX).

The cell lines 293T (CRL-3216) and THP-1 (TIB-202) were obtained from the American Type Culture Collection (ATCC). All the cell lines were authenticated by STR profiling and tested negative for mycoplasma. For osteoclast differentiation induction, the THP-1 cell line was cultured in complete medium (RPMI-1640, 100 U/ml penicillin and 100 μg/ml streptomycin, 10% FBS) and phorbol-12-myristate-13-acetate (PMA) (200 nM) for 3 days. Next, adherent cells were cultured in complete medium with RANKL (50 ng/ml) and BMSCs supernatant, and the mixed medium was refreshed every 3 days for 2 weeks.

### Rhodamine 123 and Hoechst 33342 double staining

Rhodamine 123 (C2007, Beyotime Biotechnology, Haimen, China) staining was used to determine the MMP and trace the mitochondrial network. BMSCs were counterstained with 2 μM rhodamine 123 and Hoechst 33342 for 10 min. Images were taken using a fluorescence microscope and confocal microscopy. Increased green rhodamine 123 fluorescence indicated dissipated MMP. Mitochondrial network morphology was analyzed using the mitochondrial network analysis (MiNA) toolset in ImageJ [61].

### Bioenergetic analyses/profiling

BMSCs were seeded in 96-well multiwell plates (Seahorse Bioscience, Agilent Technologies, MA, USA) and differentiated toward adipogenesis and osteogenesis for 7 days. According to a previous study [62], a mitochondrial stress test was performed by adding 10 mmol L^–1^ glucose, 1.5 μmol L^–1^ oligomycin A, 2 μmol L^–1^ FCCP, and 0.5 μmol L^–1^ Rot/Ant in succession. The basal OCR was then calculated by subtracting Rot/Ant from the unstimulated OCR value. Maximal respiration was determined following FCCP treatment. The ATP production rate was measured using an ATP rate assay (Seahorse Bioscience, Agilent Technologies, MA, USA). All the data were normalized to the CCK-8 assay.

### Statistical analysis

The results are expressed as means ± SD. Experiments were repeated independently at least three times. Statistical significance of two-group comparisons was assessed using Student’s *t* test. Analysis across multiple comparisons was performed for one-way ANOVA. To determine significance between aged and si-Neat1 mice, comparisons were made using two-way ANOVA. *p  < 0.05; **p  < 0.01; ***p < 0.001 were considered statistically significant.

Additional methodological details are provided in the Supplementary Materials.

## Supplementary information

supplementary information

Supplementary Figure 1

Supplementary Figure 2

Supplementary Figure 3

Supplementary Figure 4

Supplementary Figure 5

Supplementary Figure 6

Supplementary Figure 7

Supplementary Figure 8

Supplementary Table1

Supplementary Table2

Supplementary Table3

Supplementary Table4

Supplementary Table5

Supplementary Table6

## Data Availability

The accession numbers for the Single-Cell RNA-seq, ChIP-seq (including H3K27ac, MED1, DNase), and RNA-seq data reported in this paper are Gene Expression Omnibus (GEO): GSE113253, GSE139073.

## References

[1] Pittenger MF, Mackay AM, Beck SC, Jaiswal RK, Douglas R, Mosca JD (1999). Multilineage potential of adult human mesenchymal stem cells. Science..

[2] Discher DE, Mooney DJ, Zandstra PW (2009). Growth factors, matrices, and forces combine and control stem cells. Science..

[3] Guilak F, Cohen DM, Estes BT, Gimble JM, Liedtke W, Chen CS (2009). Control of stem cell fate by physical interactions with the extracellular matrix. Cell Stem Cell.

[4] Moerman EJ, Teng K, Lipschitz DA, Lecka-Czernik B (2004). Aging activates adipogenic and suppresses osteogenic programs in mesenchymal marrow stroma/stem cells: the role of PPAR-gamma 2 transcription factor and TGF-beta/BMP signaling pathways. Aging Cell.

[5] Li CJ, Cheng P, Liang MK, Chen YS, Lu Q, Wang JY (2015). MicroRNA-188 regulates age-related switch between osteoblast and adipocyte differentiation. J Clin Investig.

[6] Li HJ, Liu P, Xu SQ, Li YH, Dekker JD, Li BJ (2017). FOXP1 controls mesenchymal stem cell commitment and senescence during skeletal aging. J Clin Investig.

[7] Yu B, Huo LH, Liu YS, Deng P, Szymanski J, Li J (2018). PGC-1 alpha controls skeletal stem cell fate and bone-fat balance in osteoporosis and skeletal aging by inducing TAZ. Cell Stem Cell.

[8] Ambrosi TH, Scialdone A, Graja A, Gohlke S, Jank AM, Bocian C (2017). Adipocyte accumulation in the bone marrow during obesity and aging impairs stem cell-based hematopoietic and bone regeneration. Cell Stem Cell.

[9] Justesen J, Stenderup K, Ebbesen EN, Mosekilde L, Steiniche T, Kassem M (2001). Adipocyte tissue volume in bone marrow is increased with aging and in patients with osteoporosis. Biogerontology.

[10] Kawai M, Rosen CJ (2010). PPAR gamma: a circadian transcription factor in adipogenesis and osteogenesis. Nat Rev Endocrinol.

[11] McCauley LK (2010). c-Maf and you won’t see fat. J Clin Investig.

[12] Chen Q, Shou P, Zheng C, Jiang M, Cao G, Yang Q (2016). Fate decision of mesenchymal stem cells: adipocytes or osteoblasts?. Cell Death Differ.

[13] Kawai M (2013). Adipose tissue and bone: role of PPARgamma in adipogenesis and osteogenesis. Horm Mol Biol Clin Investig.

[14] Shirakawa K, Maeda S, Gotoh T, Hayashi M, Shinomiya K, Ehata S (2006). CCAAT/enhancer-binding protein homologous protein (CHOP) regulates osteoblast differentiation. Mol Cell Biol.

[15] Li Q, Gao Z, Chen Y, Guan MX (2017). The role of mitochondria in osteogenic, adipogenic and chondrogenic differentiation of mesenchymal stem cells. Protein Cell.

[16] Wang Y, Liu Y, Chen E, Pan Z (2020). The role of mitochondrial dysfunction in mesenchymal stem cell senescence. Cell Tissue Res.

[17] Geissler S, Textor M, Kuhnisch J, Konnig D, Klein O, Ode A (2012). Functional comparison of chronological and in vitro aging: differential role of the cytoskeleton and mitochondria in mesenchymal stromal cells. PLoS ONE.

[18] Correia-Melo C, Passos JF (2015). Mitochondria: Are they causal players in cellular senescence?. BBA Bioenerg.

[19] Mohammadalipour A, Dumbali SP, Wenzel PL (2020). Mitochondrial transfer and regulators of mesenchymal stromal cell function and therapeutic efficacy. Front Cell Dev Biol.

[20] Ren L, Chen X, Chen X, Li J, Cheng B, Xia J (2020). Mitochondrial dynamics: fission and fusion in fate determination of mesenchymal stem cells. Front Cell Dev Biol.

[21] Spees JL, Lee RH, Gregory CA (2016). Mechanisms of mesenchymal stem/stromal cell function. Stem Cell Res Ther.

[22] Zheng CX, Sui BD, Qiu XY, Hu CH, Jin Y (2020). Mitochondrial regulation of stem cells in bone homeostasis. Trends Mol Med.

[23] Bertolo A, Capossela S, Frankl G, Baur M, Potzel T, Stoyanov J (2017). Oxidative status predicts quality in human mesenchymal stem cells. Stem Cell Res Ther.

[24] Shum LC, White NS, Mills BN, Bentley KL, Eliseev RA (2016). Energy metabolism in mesenchymal stem cells during osteogenic differentiation. Stem Cells Dev.

[25] Wang Y, Hu SB, Wang MR, Yao RW, Wu D, Yang L (2018). Genome-wide screening of NEAT1 regulators reveals cross-regulation between paraspeckles and mitochondria. Nat Cell Biol.

[26] Clemson CM, Hutchinson JN, Sara SA, Ensminger AW, Fox AH, Chess A (2009). An architectural role for a nuclear noncoding RNA: NEAT1 RNA is essential for the structure of paraspeckles. Mol Cell.

[27] Hassan MQ, Gordon JAR, Beloti MM, Croce CM, van Wijnen AJ, Stein JL (2010). A network connecting Runx2, SATB2, and the miR-23a similar to 27a similar to 24-2 cluster regulates the osteoblast differentiation program. Proc Natl Acad Sci USA.

[28] Farmer SR (2006). Transcriptional control of adipocyte formation. Cell Metab.

[29] Boyer LA, Lee TI, Cole MF, Johnstone SE, Levine SS, Zucker JP (2005). Core transcriptional regulatory circuitry in human embryonic stem cells. Cell.

[30] Fu X, Wu S, Li B, Xu Y, Liu J (2020). Functions of p53 in pluripotent stem cells. Protein Cell.

[31] Senturk Cetin N, Kuo CC, Ribarska T, Li R, Costa IG, Grummt I (2019). Isolation and genome-wide characterization of cellular DNA:RNA triplex structures. Nucleic Acids Res.

[32] Hnisz D, Abraham BJ, Lee TI, Lau A, Saint-Andre V, Sigova AA (2013). Super-enhancers in the control of cell identity and disease. Cell.

[33] Pott S, Lieb JD (2015). What are super-enhancers?. Nat Genet.

[34] Whyte WA, Orlando DA, Hnisz D, Abraham BJ, Lin CY, Kagey MH (2013). Master transcription factors and mediator establish super-enhancers at key cell identity genes. Cell..

[35] Rauch A, Haakonsson AK, Madsen JGS, Larsen M, Forss I, Madsen MR (2019). Osteogenesis depends on commissioning of a network of stem cell transcription factors that act as repressors of adipogenesis. Nat Genet.

[36] Alvarez-Dominguez JR, Knoll M, Gromatzky AA, Lodish HF (2017). The super-enhancer-derived alncRNA-EC7/bloodlinc potentiates red blood cell development in trans. Cell Rep.

[37] Lee BK, Jang YJ, Kim M, LeBlanc L, Rhee C, Lee J (2019). Super-enhancer-guided mapping of regulatory networks controlling mouse trophoblast stem cells. Nat Commun.

[38] van Groningen T, Koster J, Valentijn LJ, Zwijnenburg DA, Akogul N, Hasselt NE (2017). Neuroblastoma is composed of two super-enhancer-associated differentiation states. Nat Genet.

[39] Rodda DJ, Chew JL, Lim LH, Loh YH, Wang B, Ng HH (2005). Transcriptional regulation of Nanog by Oct4 and Sox2. J Biol Chem.

[40] Ouyang J, Yu W, Liu J, Zhang N, Florens L, Chen JK (2015). Cyclin-dependent kinase-mediated Sox2 phosphorylation enhances the ability of Sox2 to establish the pluripotent state. J Biol Chem.

[41] Chew JL, Loh YH, Zhang W, Chen X, Tam WL, Yeap LS (2005). Reciprocal transcriptional regulation of Pou5f1 and Sox2 via the Oct4/Sox2 complex in embryonic stem cells. Mol Cell Biol.

[42] Loh YH, Wu Q, Chew JL, Vega VB, Zhang W, Chen X (2006). The Oct4 and Nanog transcription network regulates pluripotency in mouse embryonic stem cells. Nat Genet.

[43] van den Berg DL, Snoek T, Mullin NP, Yates A, Bezstarosti K, Demmers J (2010). An Oct4-centered protein interaction network in embryonic stem cells. Cell Stem Cell.

[44] Li J, Liu XY, Zuo B, Zhang L (2016). The role of bone marrow microenvironment in governing the balance between osteoblastogenesis and adipogenesis. Aging Dis.

[45] Yu B, Wang CY (2016). Osteoporosis: the result of an ‘aged’ bone microenvironment. Trends Mol Med.

[46] Eleuteri S, Fierabracci A (2019). Insights into the secretome of mesenchymal stem cells and its potential applications. Int J Mol Sci.

[47] Mardpour S, Hamidieh AA, Taleahmad S, Sharifzad F, Taghikhani A, Baharvand H (2019). Interaction between mesenchymal stromal cell-derived extracellular vesicles and immune cells by distinct protein content. J Cell Physiol.

[48] Yue R, Zhou BO, Shimada IS, Zhao Z, Morrison SJ (2016). Leptin receptor promotes adipogenesis and reduces osteogenesis by regulating mesenchymal stromal cells in adult bone marrow. Cell Stem Cell.

[49] Turner RT, Philbrick KA, Kuah AF, Branscum AJ, Iwaniec UT (2017). Role of estrogen receptor signaling in skeletal response to leptin in female ob/ob mice. J Endocrinol.

[50] Li CJ, Xiao Y, Yang M, Su T, Sun X, Guo Q (2018). Long noncoding RNA Bmncr regulates mesenchymal stem cell fate during skeletal aging. J Clin Investig.

[51] Wiley CD, Velarde MC, Lecot P, Liu S, Sarnoski EA, Freund A (2016). Mitochondrial dysfunction induces senescence with a distinct secretory phenotype. Cell Metab.

[52] Li Y, Jiang YF, Paxman J, O’Laughlin R, Klepin S, Zhu YL (2020). A programmable fate decision landscape underlies single-cell aging in yeast. Science..

[53] Lau E, Kluger H, Varsano T, Lee K, Scheffler I, Rimm DL (2012). PKC epsilon promotes oncogenic functions of ATF2 in the nucleus while blocking its apoptotic function at mitochondria. Cell..

[54] Lopez-Bergami P, Lau E, Ronai Z (2010). Emerging roles of ATF2 and the dynamic AP1 network in cancer. Nat Rev Cancer.

[55] Shah M, Bhoumik A, Goel V, Dewing A, Breitwieser W, Kluger H (2010). A role for ATF2 in regulating MITF and melanoma development. Plos Genet.

[56] Watson G, Ronai ZA, Lau E (2017). ATF2, a paradigm of the multifaceted regulation of transcription factors in biology and disease. Pharmacol Res.

[57] Jiang L, Shao C, Wu QJ, Chen G, Zhou J, Yang B (2017). NEAT1 scaffolds RNA-binding proteins and the Microprocessor to globally enhance pri-miRNA processing. Nat Struct Mol Biol.

[58] Michael AK, Grand RS, Isbel L, Cavadini S, Kozicka Z, Kempf G (2020). Mechanisms of OCT4-SOX2 motif readout on nucleosomes. Science..

[59] Chen X, Ouyang ZX, Shen Y, Liu B, Zhang Q, Wan L (2019). CircRNA_28313/miR-195a/CSF1 axis modulates osteoclast differentiation to affect OVX-induced bone absorption in mice. RNA Biol.

[60] Xu R, Shen X, Si Y, Fu Y, Zhu W, Xiao T (2018). MicroRNA-31a-5p from aging BMSCs links bone formation and resorption in the aged bone marrow microenvironment. Aging Cell.

[61] Valente AJ, Maddalena LA, Robb EL, Moradi F, Stuart JA (2017). A simple ImageJ macro tool for analyzing mitochondrial network morphology in mammalian cell culture. Acta Histochem.

[62] Tencerova M, Rendina-Ruedy E, Neess D, Faergeman N, Figeac F, Ali D (2019). Metabolic programming determines the lineage-differentiation fate of murine bone marrow stromal progenitor cells. Bone Res.

