# Supplementary material for: LncRNA NEAT1 controls the lineage fates of BMSCs during skeletal aging by impairing mitochondrial function and pluripotency maintenance
Source: Cell Death Differ. 2021 Sep 8;29(2):351–65. doi: 10.1038/s41418-021-00858-0 (PMC8816946; doi:10.1038/s41418-021-00858-0)
Supplement: Supplementary file 1 — supplementary information [file 41418_2021_858_MOESM1_ESM.docx]

**Supplementary Materials and Methods**

**Transfection of small interfering RNAs (siRNAs), microRNA mimics/inhibitors, and plasmids**

siRNAs targeting ATF2, NEAT1, and miR-27b-3p mimic/inhibitor were designed and synthesized by RiboBio (Guangzhou, China). The adenovirus vector GV315 containing NEAT1 plasmids (NR_028272) and lentivirus vector GV341 containing ATF2 plasmids (NM_001880) were purchased from GeneChem Company (Shanghai, China). siRNA and miR-27b-3p mimic/inhibitor were transfected into cells using Lipofectamine 2000 (Invitrogen, Carlsbad, CA, USA). All sequences are listed in Supplementary Table 4.

**Analysis of single-cell RNA-seq and RNA-seq**

Single-cell RNA-seq raw data generated in telomerase-immortalized human BMSCs at day 0 and day 7 of osteoblast and adipocyte differentiation were retrieved from NCBI Gene Expression Omnibus (GEO), labeling GSM3439738, GSM3439737, and GSM3439739. Sequence Read Archive (SRA) raw data were downloaded from NCBI servers. In total, the data included 2221 undifferentiated cells, 3329 cells after 7 days of osteoblast differentiation, and 7568 cells after 7 days of adipocyte differentiation. Quality controls and global-scaling normalization were performed in Seurat. The top 2000 feature genes were scaled for follow-up principal component analysis (PCA) reduction. According to JackStrawPlot and ElbowPlot, principal components were selected for tSNE and UMAP cluster analysis. Next, differentially expressed genes (DEGs) of each cluster were found by Seurat and tested using receiver operating characteristic (ROC) curves. Based on the Seurat process, Monocle was used for single-cell pseudotime ordering and identifying the root and branches in the single-cell transcriptional trajectory models.

Raw RNA-seq data generated in primary BMSCs at day 0, hour 4, day 1, day 3, and day 7 of osteoblast and adipocyte differentiation were obtained from GEO (GSE113253). Accessing from GEO (GSE139073), RNA-seq was performed on BMSCs derived from young (18 to 44 years old), middle-aged (44 to 64 years old), and aged (above 65 years old) healthy individuals. Sequence Read Archive (SRA) raw data were downloaded from NCBI servers. FastQC and hisat2 were used for quality controls and read mapping. K-means was used to cluster differentially expressed genes as determined by DESeq2. Read counts were transformed to transcripts per million (TPM) for the following gene set enrichment analysis (GSEA) and correlation analysis.

**Identification and analysis of super-enhancers (**SEs**)**

Accessing from GEO (GSE113253), DNase-seq, Med1-seq, and H3K27ac-seq analyses were performed on human BMSCs harvested at day 0, hour 4, day 1, day 3, and day 7 after the induction of osteoblast and adipocyte differentiation. Sequence Read Archive (SRA) raw data were downloaded from NCBI servers. Quality controls and read mapping were performed using FastQC and bowtie2, respectively. HOMER find-peaks were run on H3K27ac-seq with augment ‘-style histone -nfr -size 1000’ to detect peaks with specificity for histones and nucleosome free regions (Poisson p-value threshold = 0.001). Rank Ordering of SEs (ROSE) was utilized to identify and analyze SEs. DNase I hypersensitive sites were identified for DNase-seq according to the ENCODE IDR pipeline using the top 300,000 peaks identified by MACS2 from each replicate. Peaks with an IDR P-value < 0.05 were merged across all time points and used for all downstream analyses. Med1 peak calling was performed similarly to peak calling for DNase I hypersensitive sites, except that an input library (pooled input DNA from all time points) was sequenced and used as background for peak detection using MACS2. All visualization tasks were managed using Integrative Genomics Viewer (IGV).

**Gene ontology (GO) enrichment and gene set enrichment analysis (GSEA)**

GO analysis was performed using DAVID (https://david.ncifcrf.gov/home.jsp). Significantly enriched biological processes were defined as P < 0.01. Gene set enrichment analysis was performed following the GSEA (version v4.1.0) User Guide. Briefly, gene expression during osteogenesis or adipogenesis was integrated into a time series matrix, and the gene list was ranked with GSEA correlation ranking metrics. Gene sets from the Molecular Signature Database were used in the analysis to identify the pathways significantly enriched in each group. Gene sets were permutated 1000 times to obtain empirical FDR corrected p-values.

**RNA pulldown and mass spectrometry**

LncRNA NEAT1 pulldown was performed using Pierce™ Magnetic RNA-protein Pull-down Kit (#20164, Thermo, MA, USA) according to the manufacturer’s protocol. 5′Biotin-labeled oligonucleotide probes targeting the junction sites of sense and antisense NEAT1 (NR_028272) were designed and synthesized (GenePharma, Shanghai, China). A total of 10^7^ undifferentiated BMSCs were added to 200 μl of IP lysis buffer (#87787, Thermo, MA, USA), 2 μl of Halt™ Protease & Phosphatase Inhibitor Cocktail (#78445, Thermo, MA, USA), and 1μl of RNase inhibitor (200 U/ml) for cell cleavage. Next, 50 pM NEAT1 sense or antisense probe and 50 μl streptavidin magnetic beads were mixed and incubated at room temperature for 30 min with agitation. Unbound RNA was rinsed with 20 mM Tris and 100 μl of 1× RNA-protein binding buffer. Finally, 10 μl of 10× RNA-protein binding buffer, 30 μl of 50% glycine, and 30 μl of cell lysis buffer were added to streptavidin magnetic beads. After incubation at 4°C for 2 h with rotation, the streptavidin magnetic beads were rinsed with 1× washing buffer 3 times and then incubated with 50 μl elution buffer at 37°C for 30 min with agitation. Next, 50 μl elution buffer with beads were resuspended in 1× SDS loading buffer (without beta-mercaptoethanol and dithiothreitol) and boiled for 5 min. The supernatant was collected for silver staining, mass spectrometry, and Western blotting. For RNA collection, 50 μl elution buffer with beads were mixed with 800 μl of TRIzol reagent according to the manufacturer’s instructions. Detailed information about the probes sequences and antibodies is listed in Supplementary Table 5 and Table 6.

**Luciferase assays**

293T cells (CRL-3216, American Type Culture Collection) or BMSCs were seeded in 24-well plates for 24 h and then were transfected with the pGL3-basic luciferase reporter vector (GeneCopoeia, MD, USA), Renilla vector (GeneCopoeia, MD, USA), si-NEAT1, NEAT1 plasmid, miRNA control, and miR-27b-3p mimic or inhibitor using Lipofectamine 2000 (Thermo Invitrogen, MA, USA). Luciferase activities were measured 72 h after transfection using the Dual-Luciferase Reporter Assay System (GeneCopoeia, MD, USA). Firefly luciferase activity was normalized to Renilla luciferase activity for each sample. A pGL3-basic luciferase reporter vector containing the 3′-UTR fragments of BNIP3L, PPARG, BMP2K, CSF1, and NEAT1 promoter/enhancer activity was generated by GeneCopoeia.

**Chromatin immunoprecipitation (ChIP) assays**

Chromatin immunoprecipitation (ChIP) analysis was performed using EZ-Magna ChIP (#17-10086, Merck Millipore, MA, USA) according to the manufacturer’s protocol. Briefly, undifferentiated BMSCs were crosslinked with fresh 1% formaldehyde. Subsequently, the cells were lysed in SDS buffer and sonicated to shear the DNA. Lysates diluted with ChIP dilution buffer were immunoprecipitated with anti-ATF2 (CST, MA, USA) and rabbit IgG was used as an internal control. Reverse-crosslinked DNA was subjected to real-time PCR analysis. Detailed information, including the primer sequences used for real-time PCR, is listed in Supplementary Table 4.

**RNA-protein immunoprecipitation (RIP)**

The EZ-Magna RIP RNA-Binding Protein Immunoprecipitation Kit (#17-701, Merck Millipore, MA, USA) was used according to the manufacturer’s instructions. A total of 10^7^ undifferentiated BMSCs were added to 50 μl of lysis buffer, 0.25 μl of protease inhibitor cocktail, and 0.125 μl of RNase inhibitor for 5 min at 4°C. The cell lysates were incubated with beads coated with 5 μg of specific antibody and control IgG with rotation at 4°C overnight. Next, total RNA was retrieved to detect NEAT1 expression by qRT-PCR.

**Coimmunoprecipitation(coip)**

293T cells or undifferentiated BMSCs were incubated with IP Lysate Buffer (P0013, Beyotime Biotechnology, Haimen, China) supplemented with 1% Halt™ Protease & Phosphatase Inhibitor Cocktail (#78445, Thermo, MA, USA) for 30 min at 4^◦^C. For immunoprecipitation, 5 μg of specific antibody and 500 μg of protein were added to 50 μl of protein A/G agarose beads (Roche, Mannheim, Germany) with rotation overnight at 4°C. The beads were collected and washed four times with 1× washing buffer. Next, agarose beads were resuspended in 1× SDS loading buffer (without beta-mercaptoethanol and dithiothreitol) and boiled for 5 min for analysis by SDS-PAGE. Western blotting was performed using a standard protocol and the specific antibodies listed in Supplementary Table 5.

**Fluorescent in situ hybridization (FISH)**

All FISH probes were designed and synthesized by RiboBio (Guangzhou, China). NEAT1 and miR-27b-3p subcellular localization in BMSCs was examined using FISH kit (RiboBio, Guangzhou, China). Briefly, coverslips seeded with BMSCs and frozen sections of the femur were fixed with 4% paraformaldehyde for 15 min at 4°C and permeabilized with 1% Triton X-100 for 20 min. Next, the coverslips were incubated in prehybridization buffer for 30 min at 37°C. Hybridization buffer with FISH probes was added to the coverslips overnight at 37°C in the dark. Subsequently, the coverslips were washed twice with 4× SSC/0.1% Tween 20, 2× SSC, and 1× SSC for 5 min at 42°C. After staining with 4′, 6-diamidino-2-phenylindole (DAPI) for 10 min, the coverslips were detected by confocal fluorescence microscopy.

**Biotin-labeled miRNA capture**

The miRNA pulldown assay was performed using biotinylated miR-27b-3p mimic and miR-27b-3p mimic NC (GenePharma, Shanghai, China) transfected into primary BMSCs at a final concentration of 50 nM for 48 h. The biotin-coupled RNA complex was pulled down by incubating the cell lysates with streptavidin magnetic beads (#20164, Thermo, MA, USA). The enrichment of NEAT1, NEAT1_2, BMP2K, PPARG, BNIP3L, and CSF1 in bound fractions was calculated using the miR-27b-3p mimic/miR-27b-3p mimic NC ratio, which was detected by qRT–PCR.

**Flow cytometry**

Collected BMSCs were fixed with precooled 75% ethanol at 4°C overnight in the dark. After washing with phosphate-buffered saline (PBS), the cells were resuspended in 500 μl of PI/RNase A staining solution for 30 min at room temperature in the dark and were analyzed using a FACScan flow cytometer (BD Biosciences, San Jose, CA). Data analysis of the cell cycle phases (G0/G1, S, and G2/M phases) were evaluated by ModFit (Version 3.1, Verity Software House, ME, USA).

**Cell senescence-associated β-galactosidase (****SA-β-gal) staining**

BMSCs were seeded in 12-well plates and incubated with staining solution according to the SA-β-gal kit (GENMED, Shanghai, China). After 16 h of coculture, five randomly selected fields were chosen to quantify SA β-gal positive cells under inverted microscopy.

**Alizarin red staining, Oil red O staining, Trap staining, and WGA staining**

BMSCs were cultured in osteogenic induction for 10-14 days in vitro and then stained using alizarin red at room temperature for 10 minutes. Calcium deposition was identified under a light microscope. Calcified nodules were dissolved in 10% cetylpyridinium chloride, and the calcium absorbance was measured at 450 nm. After 14 days of adipogenic induction, lipid droplets were detected by Oil Red O staining. THP-1 (TIB-202, American Type Culture Collection)-induced osteoclasts were fixed with 4% paraformaldehyde and stained using a tartrate-resistant acid phosphatase (TRAP) kit (Sigma, MA, USA). More than two positively stained nuclei were identified as osteoclast-like cells. THP-1 cells were cultured on bovine bone slides to differentiate into osteoclasts. After 12 days, bone resorption pits were washed with PBS, stained with wheat germ agglutinin (WGA, 20 µg/ml) (Q12021MP, Thermo, MA, USA), and visualized using a DAB substrate kit (DA1010, Solarbio Science & Technology, Beijing, China).

**BMSCs derived Exosome isolation and identification**

To collect BMSC-derived EVs, cells reaching approximately 80%-90% confluence were washed with PBS three times and cultured in serum-free αMEM for 48 h. Next, the supernatant was harvested and centrifuged at 300 × g for 10 min to remove cell lysates, 2000 × g for 15 min to remove cell debris and 10,000 × g for 30 min to remove large EVs and collect the supernatant each time. Subsequently, the supernatant was centrifuged at 100,000 × g for 70 min twice to remove the contaminating protein, and the pellet was collected each time and resuspended in PBS. EVs that primarily contained microvesicles and exosomes in the pellet were resuspended in 100 µl of PBS and stored at -80°C. Exosomes were then identified by transmission electron microscope (TEM). Exosome protein markers were identified by Western blotting assay. The total amount of exosomes was detected by nanoparticle tracking analysis (NTA).

**Western blotting**

Western blotting was performed according to previous studies. We lysed the cells with RIPA buffer (P0013, Beyotime Biotechnology, Haimen, China) containing 1% Halt™ Protease & Phosphatase Inhibitor Cocktail (#78445, Thermo, MA, USA). Protein lysate was loaded onto 10%–15% SDS-PAGE gels and then transferred to PVDF membranes (Merck Millipore, MA, USA). Before overnight incubation with primary antibodies (Supplementary Table 5), the membranes were blocked with 5% fat-free milk for 2 h. After washing with TBST three times, the membranes were incubated for 1 h with the corresponding horseradish peroxidase-conjugated secondary antibodies (Supplementary Table 5). We observed the blots using an ECL detection kit (Merck Millipore, MA, USA) and examined the Western blotting results using ImageJ software.

**Intracellular ROS level evaluation**

BMSC samples seeded in 96 well plates (5000 cells per well) were washed with 1×PBS twice, and 100 µl of 2',7'-dichlorodihydrofluorescein diacetate (S0033, Beyotime Biotechnology, Haimen, China) probe was loaded in situ and reacted for 15 min. The excess dye was washed with PBS and the ROS level of the samples was read in a fluorescence microplate using 488 nm and 522 nm as the excitation and emission wavelengths, respectively. All the data were normalized to the Cell Counting Kit-8 (CCK-8) assay (#K1018, ApexBio Technology, TX, USA).

**Intracellular superoxide concentration detection**

Intracellular superoxide was quantitatively measured using the Superoxide Assay Kit (S0060, Beyotime Biotechnology, Haimen, China) to evaluate the intracellular oxidative stress level. BMSCs were plated into 96-well plates (5000 cells per well) and then were incubated with the Superoxide Assay Kit reagents for 3 min at 37℃. The OD450 and OD600 were monitored to quantitatively determine the superoxide levels. All the data were normalized to the CCK-8 assay.

**Oxygen consumption rate evaluation**

The oxygen consumption rate (OCR) was evaluated using a cellulate OCR Assay Kit (#BB-48211, BestBio, Shanghai, China) according to the manufacturer’s instructions. Briefly, BMSCs were seeded into 96-well plates (5000 cells per well). After osteogenic differentiation, fluorescent probes (10 μl per well) were added sequentially. Finally, oxygen mounting medium was added (2 drops per well). The OCR levels were examined every three minutes until half an hour in a fluorescence microplate reader. The excitation and emission wavelengths were 485 nm and 590 nm, respectively. All the data were normalized to the CCK-8 assay.

**Mitochondrial membrane potential (MMP) testing assay**

MMP was quantitated using an MMP assay kit with JC-1 (C2006, Beyotime Biotechnology, Haimen, China). Cells were incubated with JC-1 for 30 min. Next, aggregates and monomers of JC-1 were measured using a fluorescence microplate. The excitation and emission wavelengths of the JC-1 monomer were 490 nm and 530 nm, respectively, while JC-1 aggregates were measured using 488 nm and 522 nm as the excitation and emission wavelengths, respectively. The ratio of JC-1 aggregates to monomers was regarded as MMP. All the data were normalized to the CCK-8 assay.

**Enzyme-linked immunosorbent assay** **(ELISA)**

M-CSF, IL-6, RANKL, and TNF-α in BMSCs supernatants were analyzed using ELISA Kits for human M-CSF, IL-6, RANKL, and TNF-α (FMS-ELH045, FMS-ELH008, FMS-ELH260, and FMS-ELH036, Fcmacs, Nanjing, China) according to the manufacturer’s protocol. The absorbances at 450 nm and 630 nm were immediately measured using an automatic microplate reader after adding stop solution.

**Immunofluorescence**

Coverslips seeded with BMSCs or frozen sections of the femur were fixed with 4% paraformaldehyde and permeabilized with 1% Triton X-100. After incubation with goat serum to block nonspecific staining, primary antibodies were incubated with the cells or bone tissue overnight at 4°C. After rinsing with TBS, the cells or bone tissue were incubated with secondary IgG at 37°C for 1 h. The nuclei were stained with DAPI.

THP-1-induced osteoclasts were seeded on 24-well coverslips and cultured with DiI-stained (1,1'-dioctadecyl-3,3,3',3'-tetramethylindocarbocyanine perchlorate) (C1036, Beyotime Biotechnology, Haimen, China) BMSC supernatant for 24 h at 37°C. Next, THP-1 cells were fixed with 4% paraformaldehyde and permeabilized in PBS containing 1% Triton X-100 for 30 min at room temperature. For F-actin ring staining, THP-1 cells on creep plates were incubated with 1 ml of PBS containing 1 μl of phalloidin-TRITC conjugate (AAT-23102, AAT bioquest, CA, USA). The nuclei were labeled with DAPI at room temperature for 10 min. Fluorescent images were captured using a fluorescence microscope.

**Real-time RT-PCR**

RNA was extracted from the cells, nucleus, and cytoplasm using TRIzol reagent according to the manufacturer’s instructions. Reverse transcription was performed using 1 μg of total RNA and the PrimeScript RT reagent kit (Takara Bio, Shiga, Japan) according to the manufacturer’s protocol. Quantitative RT-PCR was performed using SYBR Green PCR Master Mix (Vazyme, Nanjing, China) and an ABI Prism 7900 Sequence detection system (Applied Biosystems, CA, USA). The levels of each mRNA or miRNA were normalized to the ACTIN, GAPDH or U6 levels. Each experiment was performed in triplicate. The primer sequences used in this study are listed in Supplementary Table 4. The 2^−ΔΔCT^ method was used to quantify the expression of the genes of interest.

**Microcomputed tomography (micro-CT) analysis and histological observation**

The microarchitectural properties of the distal femur were analyzed using a micro-CT system (Skyscan 1176, Kontich, Belgium). Bones were scanned at a high resolution (18 μm) with an energy of 70 kV and 114 μA. We applied NRecon (v1.6) and CTAn (v1.13.8.1) to reconstruct and analyze the 3D bone images. The region of interest (ROI) was defined as the area proximal to the growth plate in the distal femurs. To evaluate the trabecular bone structure, the following four parameters were calculated: the bone volume ratio (BV/TV), trabecular thickness (Tb.Th.), trabecular number (Tb.N.), and trabecular separation (Tb.Sp.).

The bone tissue was fixed with 4% paraformaldehyde for 48 h, decalcified in 10% ethylenediaminetetraacetic acid (EDTA), embedded in paraffin wax, sectioned into 4-μm-thick slices, and stained as previously described. Briefly, the bone structure was observed using HE and Masson trichrome staining, while fat accumulation was observed using oil red O staining. Quantification of osteoblasts was presented as graphs of the quantification of Ob.S/BS (osteoblast surface per bone surface) and Ob.N/BS (osteoblast number per bone surface). Quantification of adipocytes was presented as graphs of the quantification of the area and proportion of positive staining. Osteoclasts were observed by TRAP staining and quantified by Oc.N/BS (osteoclast number per bone surface) and Oc.S/BS (osteoclast number per bone surface).

**Bioluminescence capture**

After the injection of Cy5-labeled si-NEAT1, all the mice were immediately anesthetized with 2% isoflurane. One hours, three hours, six hours, nine hours, and twelve hours later, bioluminescence images were acquired using an IVIS Spectrum Xenogen Imaging System (Caliper Life Sciences).

**Fluorescence labeling analysis**

To observe the mineralizing front, the mice were administered a subcutaneous injection of 20 mg/kg of calcein (Sigma, MA, USA) 8 and 2 days before euthanasia. The undecalcified bone samples were dehydrated in 75% to 100% ethanol and were embedded in pure resin blocks after being soaked in diluted resins. The blocks were cut along the center of the femur axis to obtain 150-mm-thin sections (EXAKT, Hambach, Germany) and were further thinned between 15 and 20 mm by abrasive paper. Images were captured by confocal microscopy to study new bone formation. Histomorphometric analysis of MAR was quantified by ImageJ.

**Supplementary Table Legends**

**Supplementary Table 1.** single-cell RNA-seq-related marker gene list (logFC filter = 0.5; adjPval Filter = 0.05).

**Supplementary Table 2.** Based on the JASPAR database (<http://jaspar.genereg.net/>), transcription factor binding sites in NEAT1 promoter/enhancer sites were predicted (the relative profile score threshold was 80%).

**Supplementary Table 3.** Mass spectrometry analysis of NEAT1 binding proteins (FDR < 0.05).

**Supplementary Table 4.** List of all Potential miRNAs.

**Supplementary Table 5.** List of all prime-, probe-, and plasmid-related sequences.

**Supplementary Table 6.** Antibody list.

**Supplementary Figure Legends**

**Fig. S1. NEAT1 is an abundant marker-gene during BMSC differentiation and senescence. a** Pseudotime ordering of single cells using 2D PCA. The data point represents a single cell colored by the clusters collected in Fig. 1B (left panel). The data point represents a single cell colored by cell type collected (middle panel). **b** Violin plots of related differentiation induction from ten clusters. **c** Transcript information of NEAT1 in humans and mice. **d** Heatmap of the top 20 differentiated genes of ten clusters. **e** Correlation analysis of NEAT1 expression based on RNA-seq. **f** qRT-PCR analysis of the levels of NEAT1_2 expression in young and aged human BMSCs (n = 3). **g** qRT-PCR analysis of the levels of Neat1 and Neat1_2 expression in BMSCs derived from young and aged mice (n = 3). **h** qRT-PCR analysis of the levels of NEAT1_2 expression in human BMSCs during osteogenesis and adipogenesis. **i-j** Abundance of NEAT1 in the cytoplasm or nucleus during human BMSC aging and differentiation (Y: young, M: middle-aged, O: aged, n = 3). **k** qRT-PCR analysis of the expression levels of nuclear NEAT1 in young and aged human BMSCs (n = 3). **l** qRT-PCR analysis of the expression levels of cytoplasmic NEAT1 in young and aged human BMSCs (n = 3). **m** Using FISH assay, localization of NEAT1 in human BMSCs during osteogenic differentiation was detected. Scale bar: 20 μm. The results were presented as means ± S.D. **p < 0.01 by Student’s t test and one-way ANOVA.

**Fig. S2. SE-associated NEAT1 is activated by ATF2. a** Hockey stick plots showing the rank order of H3K27ac signals for all enhancers in BMSCs during osteogenic differentiation. Inserted panels showing selected GO functional categories of SE-associated genes. **b** Integrative Genomics Viewer (IGV) showing H3K27ac-seq together with the DNase-seq and Med1-seq read density in NEAT1 during BMSC osteogenic and adipogenic differentiation. **c** Venn diagram showing TFBS from the promoter, enhancer site 1, and enhancer site 2. **d** ATF2 protein expression level of BMSCs transfected with normal control or ATF2 small interfering (si-ATF2) (left panel) and control vector or ATF2 plasmid (right panel).

**Fig. S3. NEAT1 regulates the lineage fates of BMSCs by impairing mitochondrial function. a** Rhodamine staining and related fluorescence quantification in young and aged BMSCs. **b** qRT-PCR analysis of NEAT1 expression levels in BMSCs transfected with normal control or si-NEAT1 and **c** control vector or NEAT1 plasmid. **d** Fluorescence intensity of rhodamine (left panel) and branch length of the mitochondrial morphological skeleton during aged BMSCs transferred with si-NEAT1. **e** Fluorescence intensity of rhodamine (left panel) and branch length of the mitochondrial morphological skeleton during young BMSCs transferred with the NEAT1 plasmid. **f** Representative EM micrographs. The arrows indicate representative mitochondria. **g** si-NEAT1 decreased BNIP3L expression as shown by qRT-PCR. **h** The NEAT1 plasmid enhanced BNIP3L expression as demonstrated by qRT-PCR. Scale bars: 50 μm (a); 1 μm (f). The results were presented as means ± S.D. *p < 0.05; **p < 0.01; #p > 0.05 by Student’s t test and one-way ANOVA.

**Fig. S4. NEAT1 attenuates BMSC pluripotency by scaffolding SOX2 and CDK2. a-b** Expression levels of NEAT1 detected by qRT-PCR after CDK2 and OCT4 immunoprecipitation in BMSCs. **c** CatRAPID signature module prediction of the RNA-binding propensity for OCT4 followed by prediction of RNA-binding regions. **d** CatRAPID fragment module prediction of the interaction profile and matrix between the OCT4 and NEAT1. **e-f** Multiple labeling of SOX2, OCT4, and CDK2 immunofluorescence and NEAT1 FISH in young and aged BMSCs. **g** CDK2 and Myc immunoprecipitated using an anti-Flag antibody. **h** Western blotting detection of SOX2 protein expression in BMSCs transfected with normal control and si-SOX2. **i** Western blotting showed NEAT1 pulldown of phosphorylated SOX2 (Ser249/250). **j-k** Western blotting detection of CDK2 protein expression in BMSCs transfected with normal control, si-CDK2, and CDK2 plasmids. **l** SA-β-gal staining, cell cycle detection, and related quantification were applied to young and aged BMSCs (n = 3). **m** The expression levels of cell cycle distribution (CCNE1 and CCND1) and cellular senescence (P53, P21, and P16)-related proteins in young and aged BMSCs are detected by Western blotting. **n** SA-β-gal staining, cell cycle detection, and related quantification were applied to aged BMSCs transfected with si-NEAT1. **o** Detection of cell cycle distribution and cellular senescence-related proteins in aged BMSCs transfected with si-NEAT1. **p-q** SA-β-gal staining, cell cycle detection, and Western blotting of related proteins were applied to BMSCs with NEAT1 overexpression. Scale bars: 20 μm (e and f); 200 μm (l, n, and p). The results were presented as means ± S.D. *p < 0.05; #p > 0.05 by Student’s t test and one-way ANOVA.

**Fig. S5. NEAT1 attenuates osteogenesis in aged BMSCs. a** Alizarin red staining was applied to aged BMSCs transfected with si-NEAT1. **b** Alizarin red staining was applied to young BMSCs transfected with the NEAT1 plasmid. Scale bar: 200 μm.

**Fig. S6. NEAT1 promotes paracrine CSF1-dependent osteoclastic activation. a** The concentrations of M-CSF, TNF-α, IL-6, and RANKL in young, middle-aged, and aged BMSCs supernatants were detected by ELISA (n = 5). **b** Quantification of TRAP^+^ cells during THP-1 differentiation. **c** Quantification of WGA^+^ area in the bone resorption assay. **d** A model for CSF1 delivery from BMSCs to THP-1 cells via the paracrine pathway and EVs.

**Fig. S7. NEAT1 serves as a miR-27b-3p sponge and mediates the characteristics of aged BMSCs. a** qRT-PCR analysis of the levels of miR-27b-3p expression in BMSCs during osteogenic differentiation. **b** si-NEAT1 enhanced miR-27b-3p expression as demonstrated by qRT-PCR. **c** The NEAT1 plasmid decreased miR-27b-3p expression as demonstrated by qRT-PCR. **d** qRT-PCR detection of miR-27b-3p expression in the NEAT1 pulldown assay. **e** qRT-PCR detection of the expression of NEAT1, NEAT1_2, BMP2K, PPARG, BNIP3L, and CSF1 in the miR-27b-3p pulldown assay. **f** Expression levels of NEAT1 detected by qRT-PCR after AGO2 immunoprecipitation in BMSCs. **g** Western blotting detection of AGO2 expression in the NEAT1 pulldown assay. **h** Western blotting revealed that the miR-27b-3p mimic rescued the downregulation of ALPL, RUNX2, and OPN by NEAT1 overexpression. **i** Western blotting showed that the miR-27b-3p inhibitor decreased the upregulation of ALPL, RUNX2, and OPN by si-NEAT1. The results were presented as means ± S.D. *p < 0.05; **p < 0.01; #p > 0.05 by Student’s t test and one-way ANOVA.

**Fig. S8. si-Neat1 delivery prevents bone loss and marrow fat accumulation in aged mice. a** Schematic diagram illustrating the experimental design of si-Neat1 treatment in aged mice (n = 5). **b** Bioluminescence detection of si-NEAT1 distribution and maintenance in mice. **c** Overview of the main organs from normal control, si-NC-, and si-Neat1-treated mice. **d** qRT-PCR detection of the expression levels of NEAT1, miR-27b-3p, and target genes in BMSCs derived from aged mice with si-NEAT1 treatment. **e** Double-labeling of immunofluorescence of Nestin and Neat1 FISH were applied to frozen sections of the mouse femur. **f** Oil red O staining of the middle femur indicated that adipogenesis was decreased in aged mice with si-Neat1 treatment. **g** H&E staining of main organs from normal control, si-NC-, and si-Neat1-treated mice. Scale bars: 50 μm (e and f); 200 μm (g). The results were presented as means ± S.D. *p < 0.05; **p < 0.01; #p > 0.05 by Student’s t test and one-way ANOVA.
