# Supplementary figures and images for: LncRNA NEAT1 controls the lineage fates of BMSCs during skeletal aging by impairing mitochondrial function and pluripotency maintenance
Source: Cell Death Differ. 2021 Sep 8;29(2):351–65. doi: 10.1038/s41418-021-00858-0 (PMC8816946; doi:10.1038/s41418-021-00858-0)

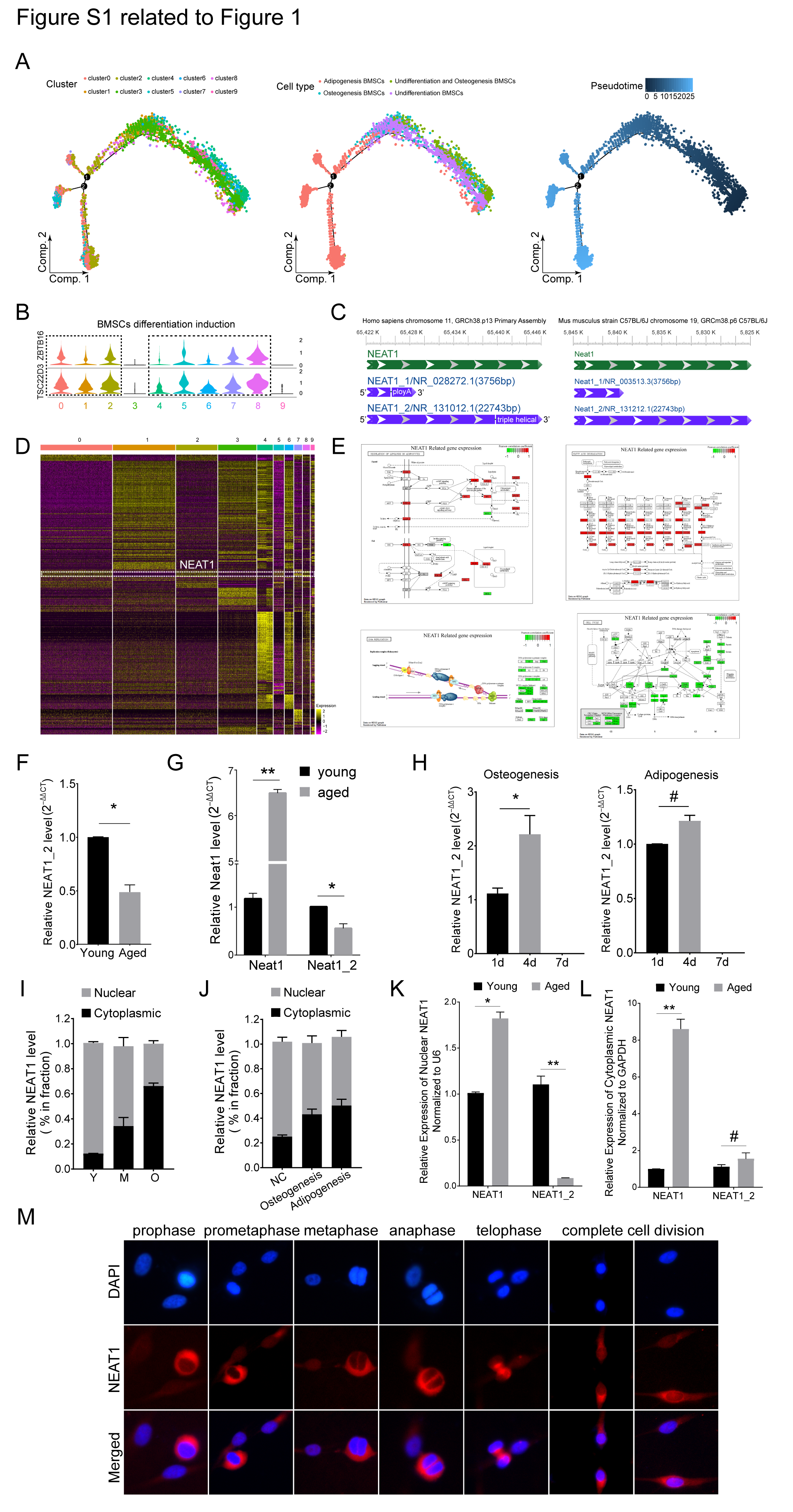

Supplement: Supplementary file 2 — Supplementary Figure 1 [file 41418_2021_858_MOESM2_ESM.tif]

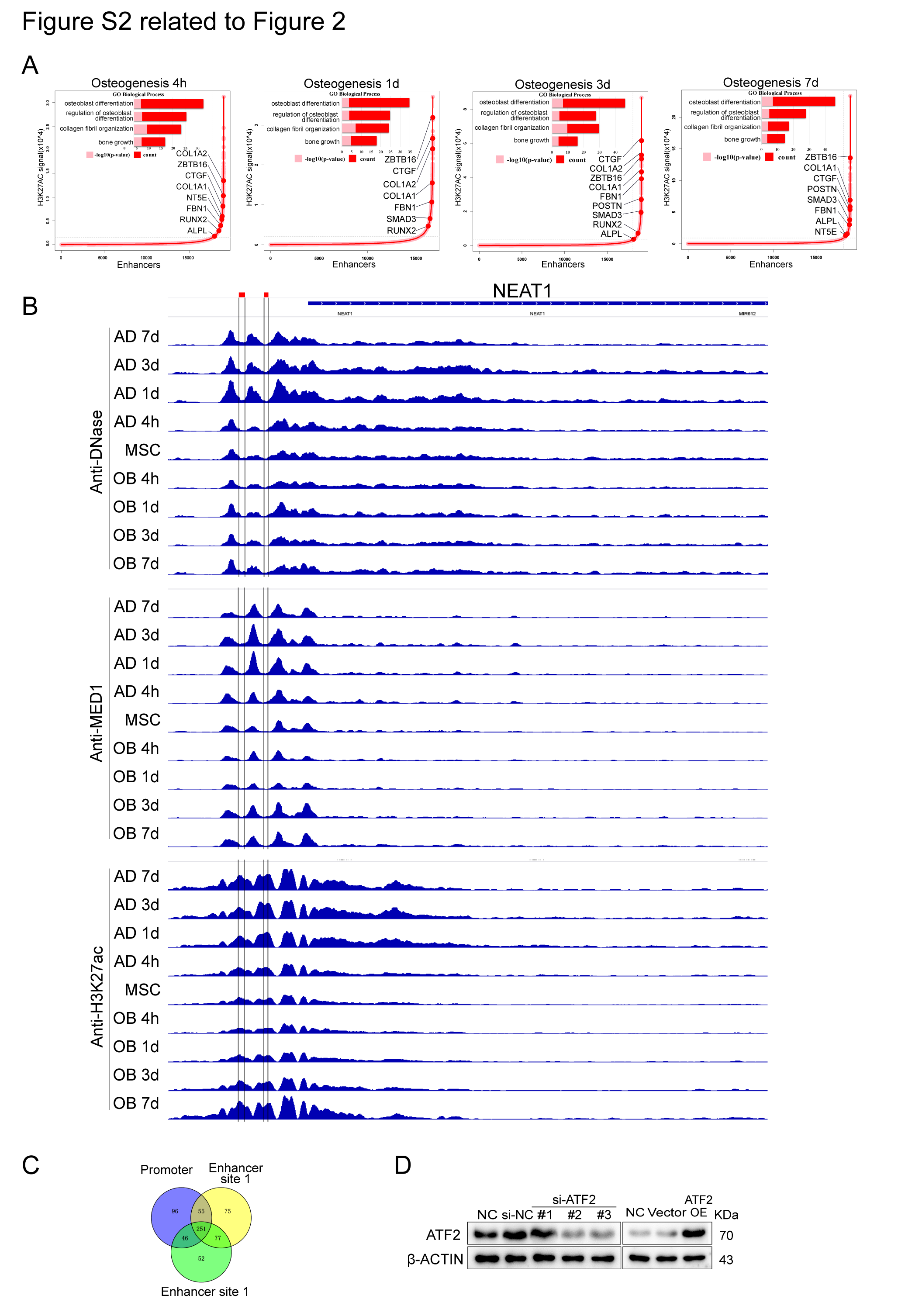

Supplement: Supplementary file 3 — Supplementary Figure 2 [file 41418_2021_858_MOESM3_ESM.tif]

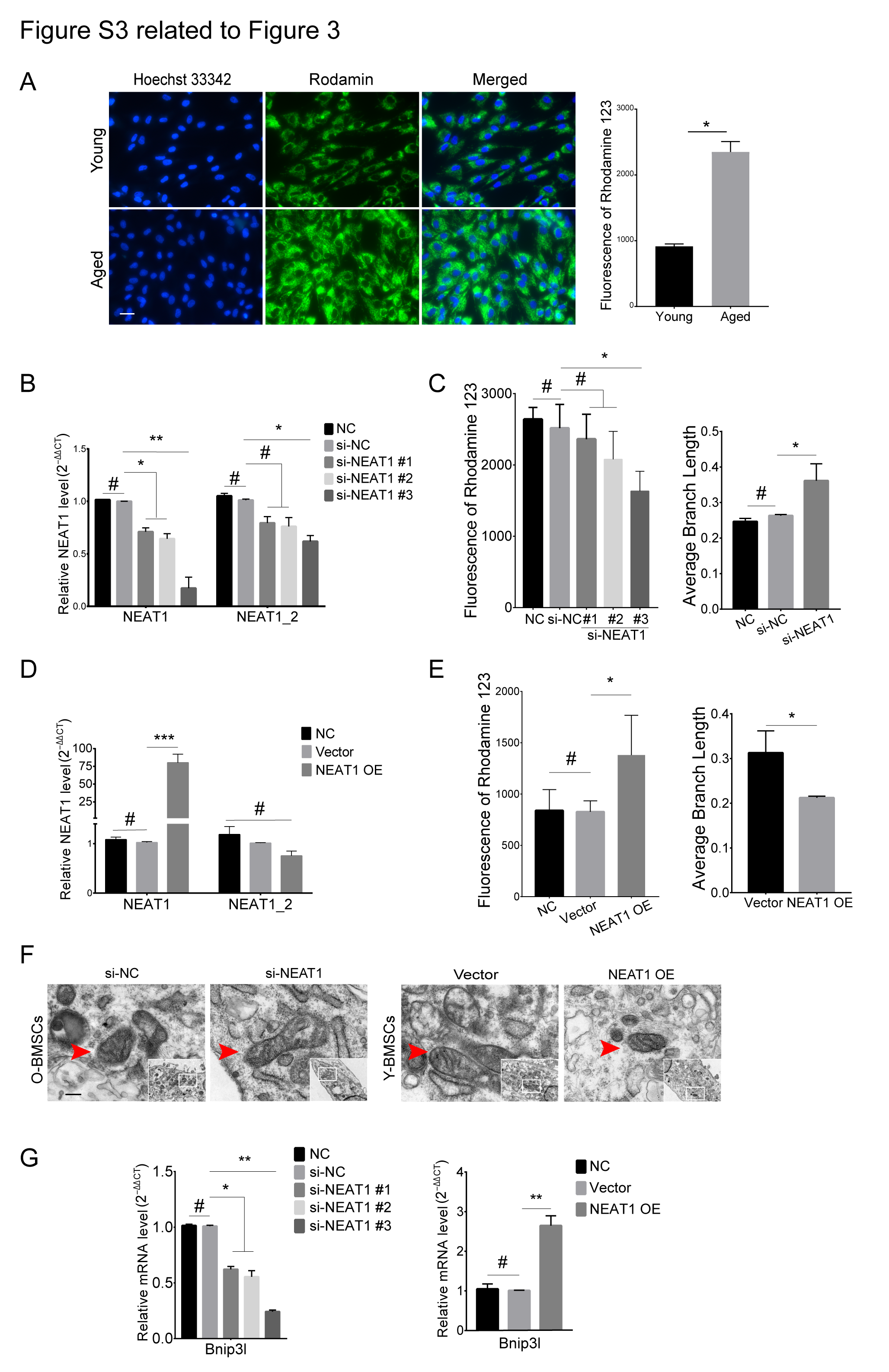

Supplement: Supplementary file 4 — Supplementary Figure 3 [file 41418_2021_858_MOESM4_ESM.tif]

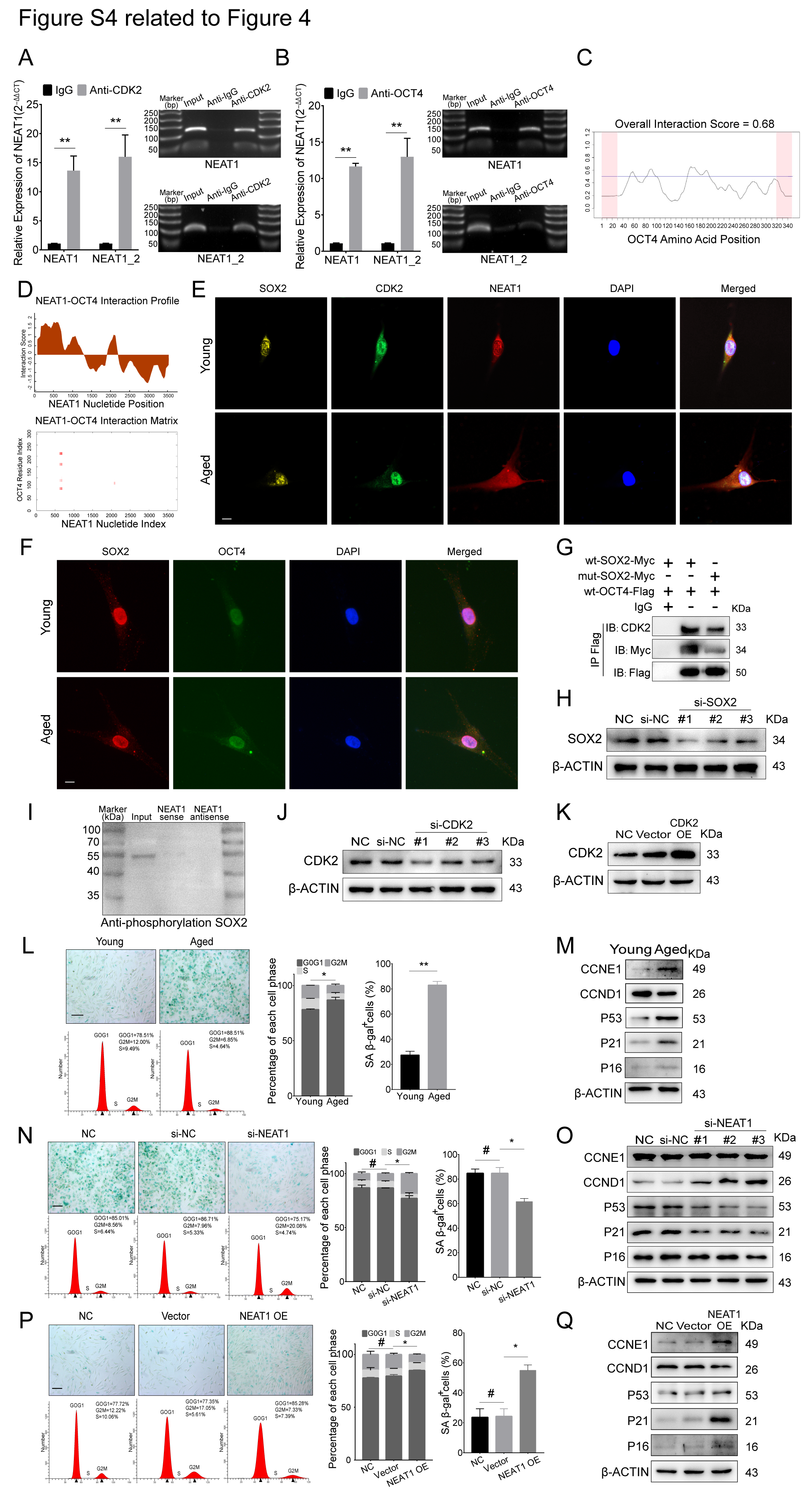

Supplement: Supplementary file 5 — Supplementary Figure 4 [file 41418_2021_858_MOESM5_ESM.tif]

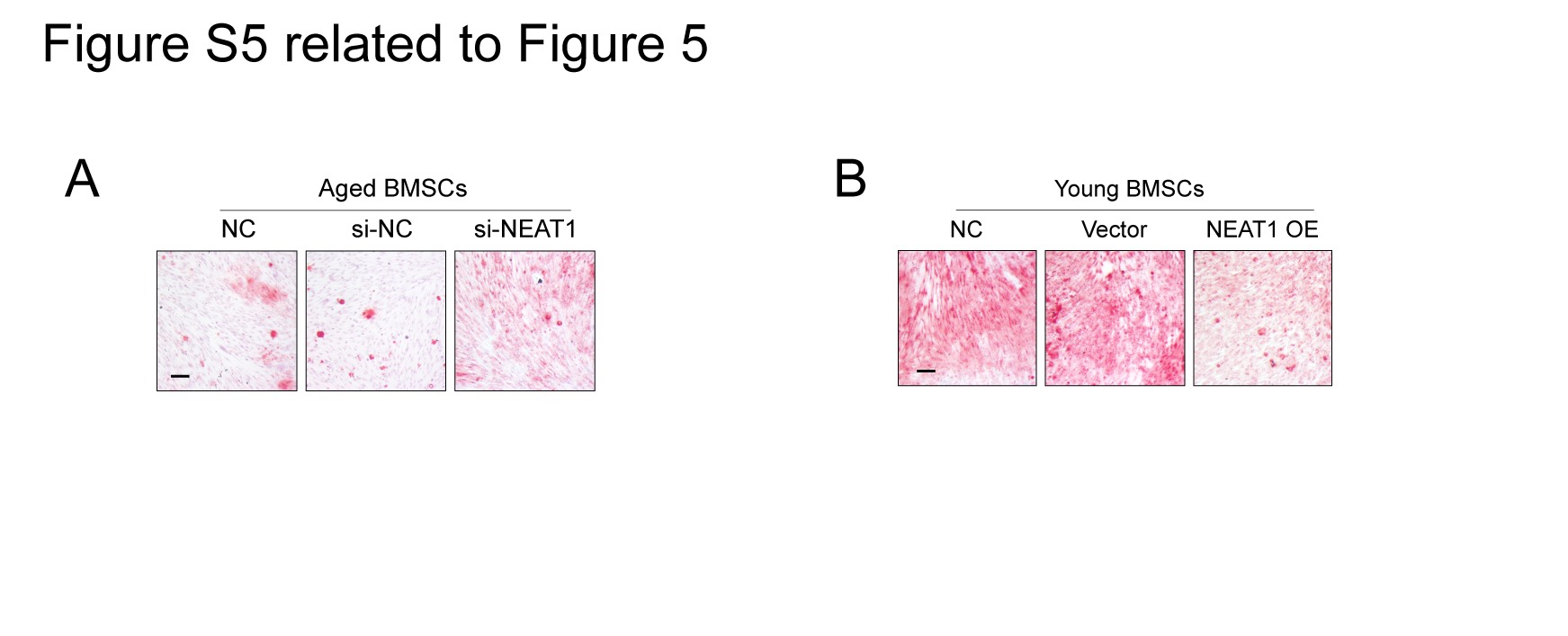

Supplement: Supplementary file 6 — Supplementary Figure 5 [file 41418_2021_858_MOESM6_ESM.tif]

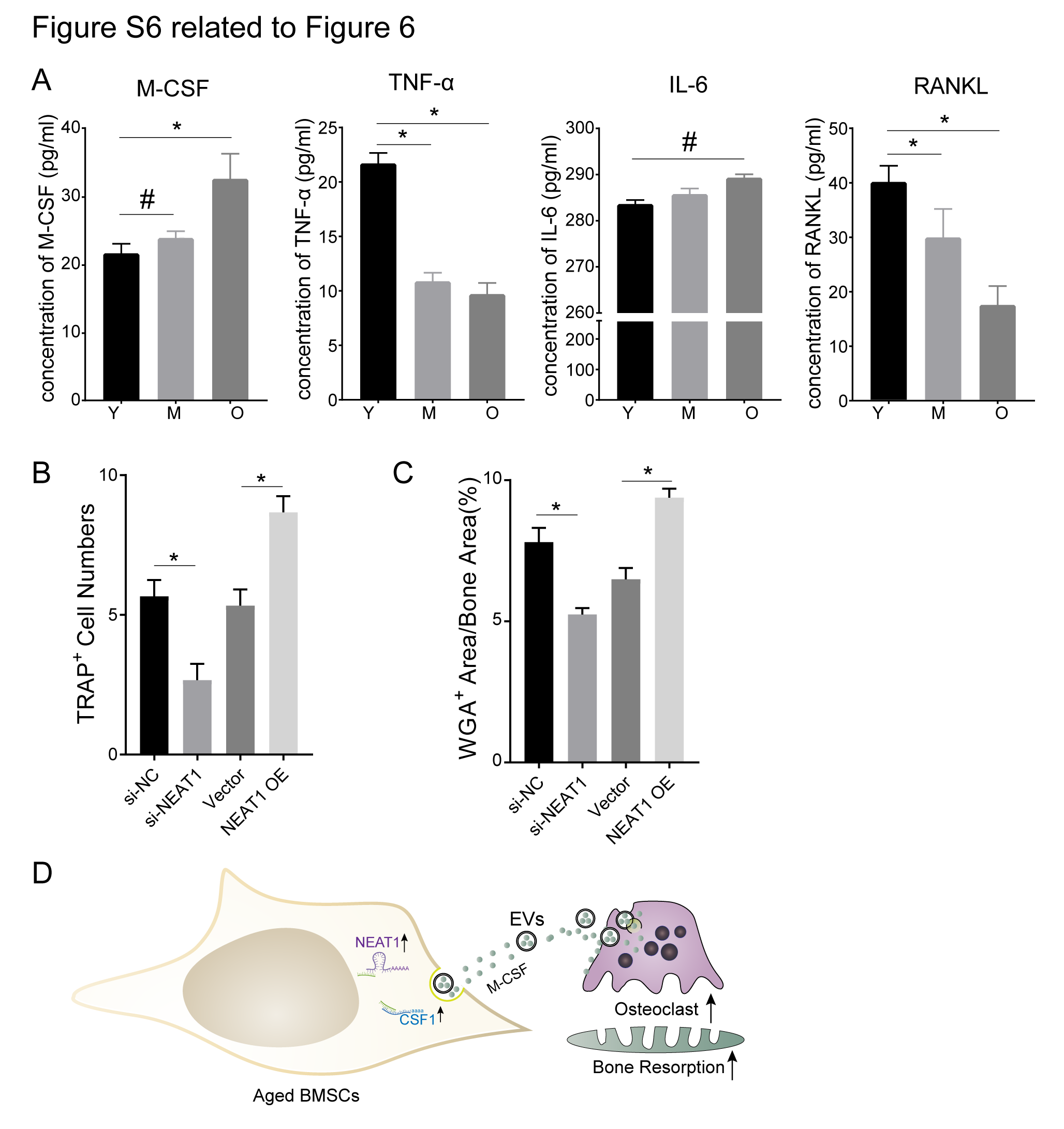

Supplement: Supplementary file 7 — Supplementary Figure 6 [file 41418_2021_858_MOESM7_ESM.tif]

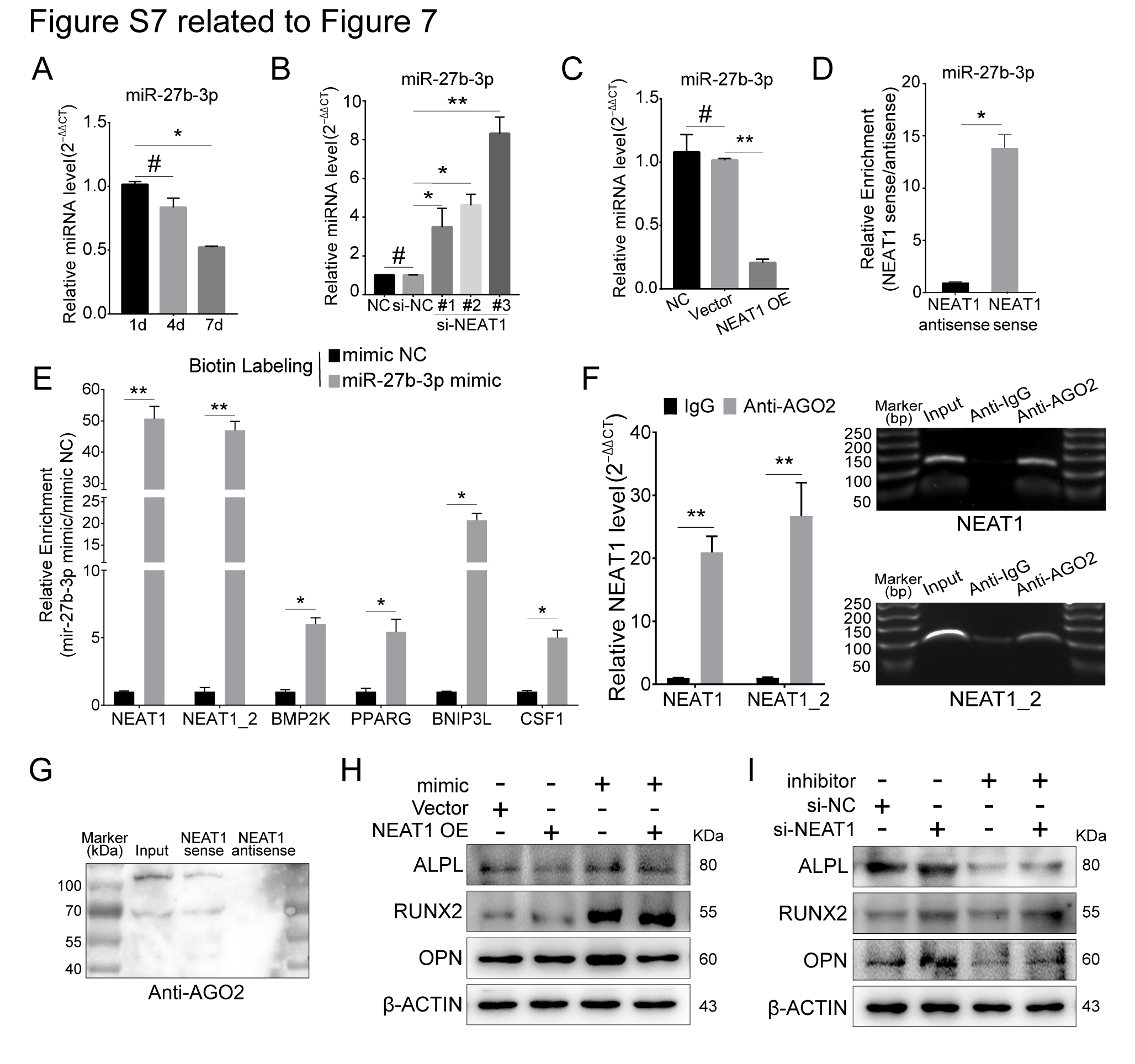

Supplement: Supplementary file 8 — Supplementary Figure 7 [file 41418_2021_858_MOESM8_ESM.tif]

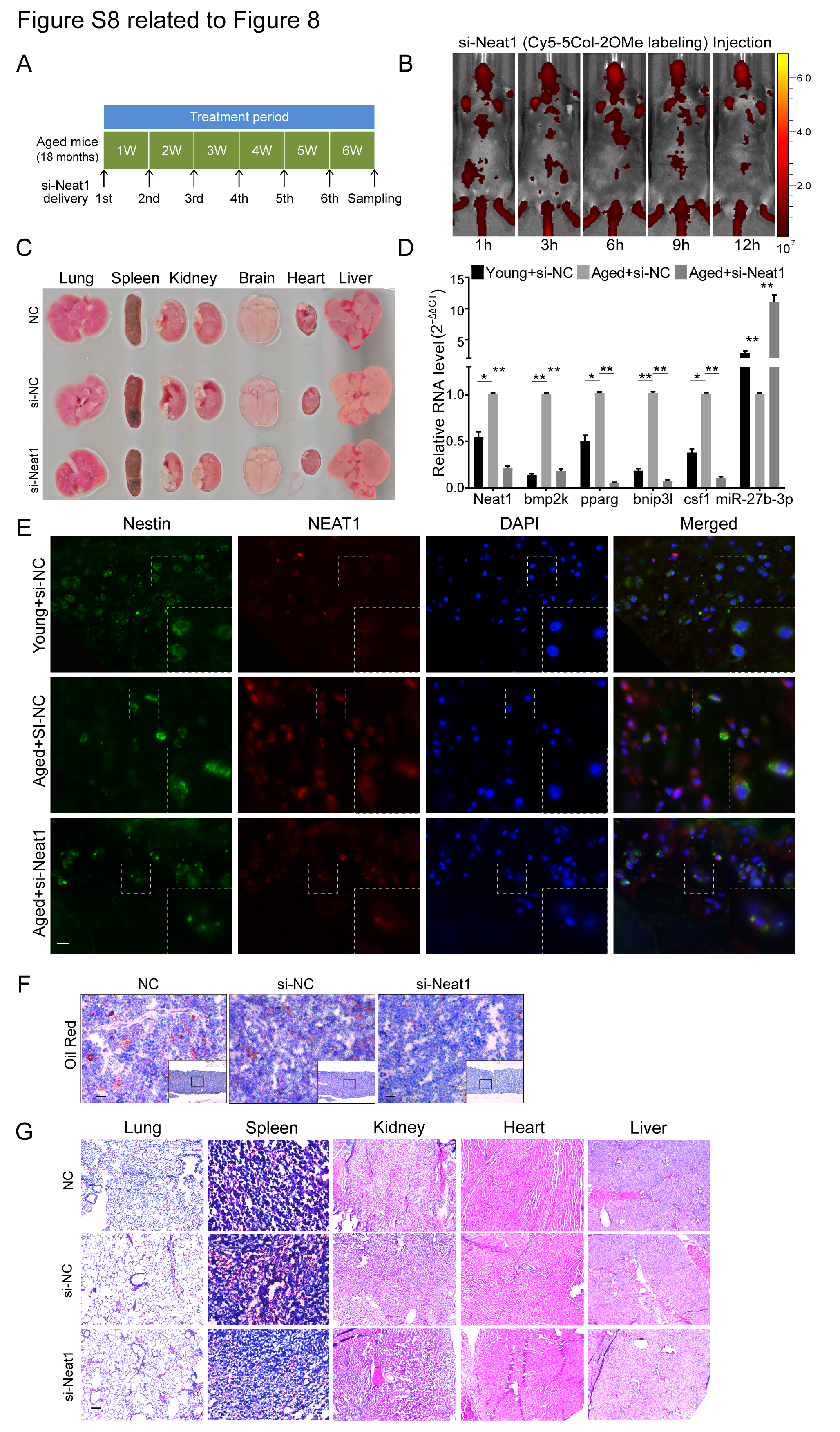

Supplement: Supplementary file 9 — Supplementary Figure 8 [file 41418_2021_858_MOESM9_ESM.tif]
